# Endothelin-1 induces Zfp36 family RNA-binding proteins and restrains cytokine and chemokine production in reactive astrocytes

**DOI:** 10.1016/j.jbc.2026.111424

**Published:** 2026-04-02

**Authors:** Yutaka Koyama, Aina Nishiuma, Nagi Takahashi, Eri Izumikawa, Chisato Hamada, Yasuhiko Izumi, Shigeru Hishinuma, Shotaro Michinaga

**Affiliations:** 1 Laboratory of Pharmacology, Kobe Pharmaceutical University, Kobe, Japan; 2 Department of Pharmacodynamics, Meiji Pharmaceutical University, Kiyose, Tokyo, Japan

**Keywords:** astrocyte, cytokine/chemokine, endothelin, RNA-binding protein, traumatic brain injury

## Abstract

Zinc-finger protein 36 (Zfp36) family RNA-binding proteins, such as tristetraprolin (TTP/Zfp36), butyrate response factor (BRF)-1/Zfp36L1, and BRF-2/Zfp36L2, regulate the expression of cytokine/chemokine mRNA with AU-rich elements. In traumatic brain injury (TBI), reactive astrocytes produce various cytokines and chemokines that induce neuroinflammation. However, despite their importance in neuroinflammation, little is known about the regulation of cytokine and chemokine production by the Zfp36 family proteins in astrocytes. Endothelin-1 (ET-1), which promotes the conversion to reactive astrocytes, stimulates astrocytic cytokine and chemokine production. In the present study, we examined the effects of ET-1 on Zfp36 family protein expression in astrocytes and the roles of these proteins in cytokine/chemokine production. ET-1 (100 nM) increased the expression of TTP and BRF-1 in cultured astrocytes. In a mouse model of TBI, expression of TTP and BRF-1 increased, which was reduced by intracerebroventricular administration of BQ788, an ET_B_ antagonist. Immunohistochemical analyses showed that TTP and BRF-1 were present in reactive astrocytes. Knockdown of TTP by siRNA enhanced the production of ET-induced CCL2 and IL-6 in cultured astrocytes, while BRF-1 knockdown enhanced the CCL2, CXCL1, and CX3CL1 production. RNA immunoprecipitation/PCR analyses showed that ET-1 stimulated TTP binding to CCL2 and IL-6 mRNAs, and BRF-1 binding to CCL2, CXCL1, and CX3CL1 mRNAs. These results suggest that ET-1 stimulates the induction of TTP and BRF-1 in astrocytes and that the production of some astrocytic chemokine/cytokine is negatively regulated by the increments in TTP and BRF-1 production.

Neuroinflammation following traumatic brain injury (TBI) and stroke is a critical process that causes secondary neural tissue damage from the injured core to the surrounding regions. The pathophysiological responses leading to neuroinflammation in the damaged brain include glial cell activation, increased permeability of brain microvessels, infiltration of blood inflammatory cells into the brain, and neuronal degeneration (1, 2). These pathophysiological responses are typically induced by excess production of cytokines and chemokines. In brain disorders, astrocytes produce various cytokines and chemokines (3, 4, 5, 6, 7, 8). In the acute phase of TBI and stroke, resting astrocytes turn their phenotype into reactive astrocytes, and this phenotypic change is accompanied by increased production of astrocytic bioactive substances. Since the increased production of chemokines and cytokines by reactive astrocytes induces pathophysiological responses leading to neuroinflammation (9, 10, 11), regulation of astrocytic function has been proposed to be a therapeutic target for TBI and stroke. Therefore, investigating the regulatory mechanisms underlying astrocytic cytokine/chemokine production in brain disorders is important for establishing new treatments for acute brain injury.

Investigations of immune cells have revealed post-transcriptional regulation of mRNA expression by RNA-binding proteins (RBPs) (12). The 3′-untranslated regions (UTRs) of several cytokine/chemokine mRNAs have a common nucleotide sequence called an AU-rich element (ARE), which some types of RBPs recognize. Zinc-finger protein 36 (Zfp36) family RBPs, such as tristetraprolin (TTP/Zfp36), butyrate response factor (BRF)-1/Zfp36L1, and BRF-2/Zfp36L2, bind to AREs in the mRNA and cause mRNA degradation, reducing the functions of some chemokines and cytokines (10, 13, 14). Induction mechanisms of the Zfp36 family of proteins have been extensively investigated in inflammatory cells (13, 14). These studies have shown that the activation of Toll-like and T-cell receptors increases TTP and BRF-1 expression, although it stimulates cytokine/chemokine production (15, 16, 17, 18). Since excessive cytokine/chemokine production results in tissue damage, the induction of Zfp36 family proteins is proposed to be an intracellular negative-feedback mechanism. On the other hand, as for nerve tissue, a few reports showed upregulation of TTP in animal models of cerebral hemorrhage and brain ischemia (19, 20). In addition, Astakhova *et al*. (21) observed TTP in cultured astrocytes. However, little is known about the regulatory mechanisms underlying the expression of Zfp36 family RBPs, including TTP, in acute brain injury, and their roles in cytokine/chemokine production in astrocytes.

Endothelin-1 (ET-1) is a vasoconstrictor peptide that is expressed in the brain. Brain ET-1 production increases in TBI and stroke, inducing several pathophysiological responses. ET-1 receptors are classified into ET_A_ and ET_B_ types, and ET_B_ receptors are highly expressed in astrocytes (22). Studies in brain injury models have shown that stimulation of astrocytic ET_B_ receptors promotes phenotypic changes to reactive astrocytes (23, 24). The production of some chemokines in reactive astrocytes was increased by the administration of an ET_B_ agonist in rats (25). In cultured astrocytes, ET-1 increased the production of various cytokines/chemokines, suggesting that ET-1 stimulates astrocytes to induce neuroinflammation (26, 27, 28). Our previous studies using a mouse model of TBI showed that the induction of reactive astrocytes and disruption of the blood–brain barrier (BBB) were reduced by the administration of an ET_B_ antagonist (22, 29) In the present study, to clarify the mechanisms underlying cytokine and chemokine production in astrocytes, the effects of ET-1 on the expression levels of Zfp36 family proteins in cultured astrocytes and a TBI animal model were examined. The results showed that ET-1 increased astrocytic TTP and BRF-1 expression through the ET_B_ receptors. Furthermore, inhibition of TTP and BRF-1 enhanced the expression of astrocytic CCL2/MCP-1, CXCL1/GROα, CX3CL1/fractalkine, and interleukin (IL)-6.

## Results

### ET-1 increased the expression of TTP and BRF-1 in cultured rat astrocytes

**Figure 1:**
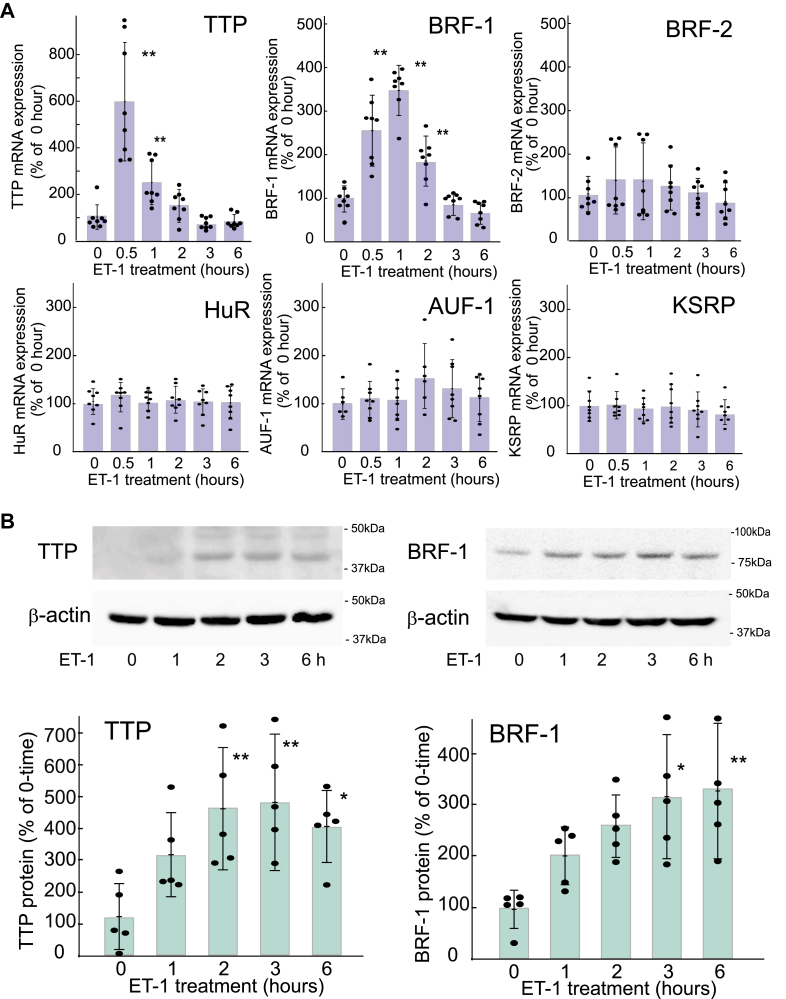
**Effects of ET-1 on the expression of astrocytic RBP**. *A*, mRNA expression. Cultured astrocytes were treated with 100 nM ET-1 for the durations indicated. The expression levels of RBP mRNA were normalized to that of G3PDH. Results are presented as mean ± SD of the findings for six to eight different mRNA preparations. Shapiro–Wilk test showed that the data for all groups were normally distributed (*p* = 0.0623–0.9963). Therefore, one-way ANOVA was applied [TTP;*F* (6,65) = 26.45, *p* = 1.062 × 10^-15^, BRF-1;*F* (5,42) = 39.17, *p* = 9.165 × 10^-15^]. ∗*p* < 0.05, ∗∗*p* < 0.01 *versus* no ET-1 treatment (0 h) by Dunnett test. *B*, TTP and BRF-1 protein expression. Cultured astrocytes were treated with 100 nM ET-1 for 1 to 6 h. Protein levels of TTP and BRF-1 were normalized to that of β-actin. Results are presented as mean ± SD of the findings for five different protein preparations. Shapiro–Wilk test showed that the data for all groups were normally distributed (*p* = 0.1220–0.9781). Therefore, one-way ANOVA was applied [TTP;*F* (4,20) = 9.812, *p* = 0.0181, BRF-1;*F* (4,20) = 3.485, *p* = 0.0257]. ∗*p* < 0.05, ∗∗*p* < 0.01 *versus* no ET-1 treatment (0 h) by o Dunnett test.

**Figure 2:**
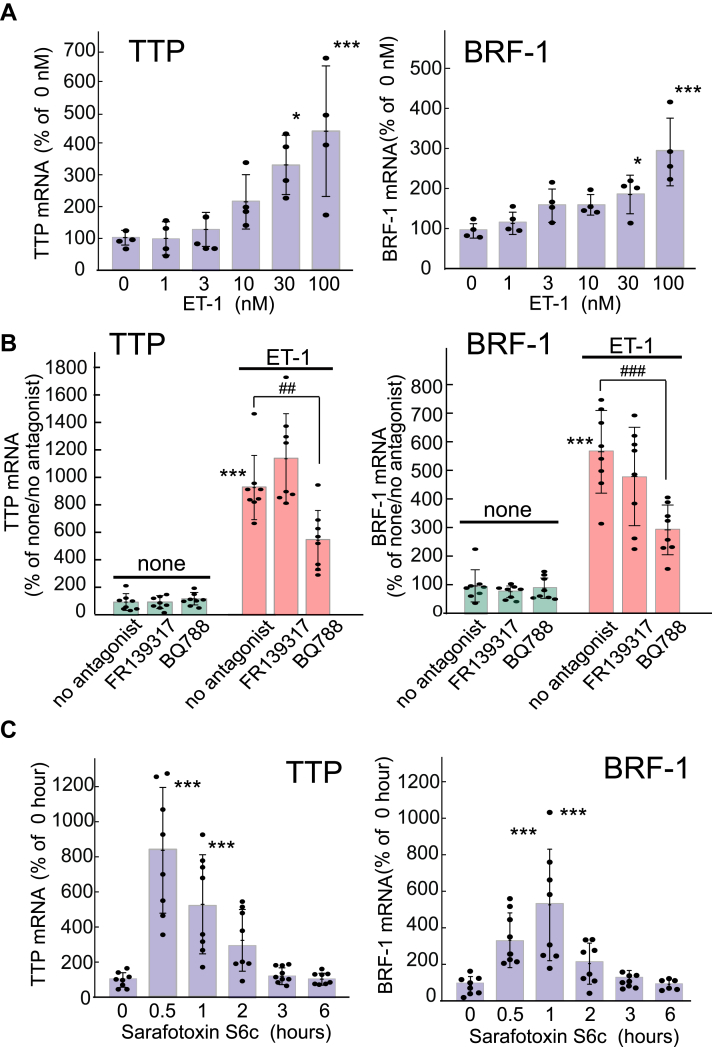
**Characterization of ET-induced astrocytic TTP and BRF-1 expression**. *A*, Dose dependence of ET-induced TTP and BRF-1 mRNA expression. Cultured astrocytes were treated with ET-1 at the concentrations indicated. TTP and BRF-1 mRNA levels were determined 0.5 and 1 h after the ET-1 addition, respectively. Results are presented as mean ± SD of the findings for four different mRNA preparations. Shapiro–Wilk test showed that the data for all groups were normally distributed (*p* = 0.0942–0.9779). Therefore, one-way ANOVA was applied [TTP;*F* (5,18) = 6.993, *p* = 0.0448, BRF-1;*F* (5,18) = 9.167, *p* = 1.792 × 10^-4^]. ∗*p* < 0.05, ∗∗∗*p* < 0.001 *versus* 0 nM ET-1 by Dunnett test. *B*, Effects of ET receptor antagonists on the ET-1-induced increments in astrocytic TTP and BRF-1 expression. Cultured astrocytes were treated with 100 nM ET-1 in the presence or absence of 1 μM BQ788 and 1 μM FR139317. TTP and BRF-1 mRNA levels were determined 0.5 and 1 h after the ET-1 addition, respectively. Results are presented as mean ± SD of the findings for eight different mRNA preparations. Shapiro–Wilk test showed that the data for all groups were normally distributed (*p* = 0.0674–0.8682). Therefore, one-way ANOVA was applied [TTP;*F* (5,42) = 47.46, *p* = 3.281 × 10^-16^, BRF-1;*F* (5,42) = 34.90, *p* = 6.413 × 10^-14^]. ∗∗∗*p* < 0.001 *versus* none/no antagonist, ^##^*p* < 0.01, ^###^*p* < 0.001 *versus* ET-1/no antagonist by Tukey test. *C*, Effects of sarafotoxin S6c on astrocytic TTP and BRF-1 expression. Cultured astrocytes were treated with 100 nM sarafotoxin S6c for the indicated durations. The expression levels of TTP and BRF-1 mRNAs were normalized to that of G3PDH. Results are presented as mean ± SD of the findings for six to eight different mRNA preparations. Shapiro–Wilk test showed that the data for all groups were normally distributed (*p* = 0.0566–0.8293). Therefore, one-way ANOVA was applied [TTP;*F* (5,40) = 10.09, *p* = 2.641 × 10^-6^, BRF-1;*F* (5,40) = 17.00, *p* = 3.604 × 10^-9^]. ∗∗*p* < 0.01, ∗∗∗*p* < 0.001 *versus* no sarafotoxin S6c treatment (0 h) by Dunnett test.

The effect of ET-1 on the expression of astrocytic RBPs of the Zfp36 family was examined. Treatment of cultured rat astrocytes with ET-1 (100 nM) for 0.5 to 2 h increased TTP and BRF-1 mRNA levels (Fig. 1*A*), but the increased expression rapidly declined with further treatments. The expression levels of the other RBPs recognizing AREs (BRF-2, ARE factor-1 [AUF-1], human antigen R [HuR], and KH-type splicing regulatory protein [KSRP]) were not affected by ET-1 treatment for 0.5 to 6 h (Fig. 1*A*). Immunoblot analysis showed that ET-1 increased the TTP and BRF-1 protein levels in cultured astrocytes (Fig. 1*B*). Treatment with ET-1 did not affect TTP or BRF-1 expression in the cultured cerebral neurons or microglia (Fig. S1). ET-1 increased the expressions of astrocytic TTP and BRF-1 in a dose-dependent manner, where significant increases observed at concentrations above 30 nM (Fig. 2*A*). The effects of ET-1 (100 nM) on TTP and BRF-1 mRNA expression levels were reduced by the ET_B_ receptor antagonist BQ788 (1 μM), but not by the ET_A_ receptor antagonist FR139317 (1 μM) (Fig. 2*B*). The selective ET_B_ receptor agonist sarafotoxin S6c (100 nM) increased TTP and BRF-1 mRNA levels in cultured astrocytes (Fig. 2*C*).

### Expression levels of RBPs in the FPI-induced TBI model

**Figure 3:**
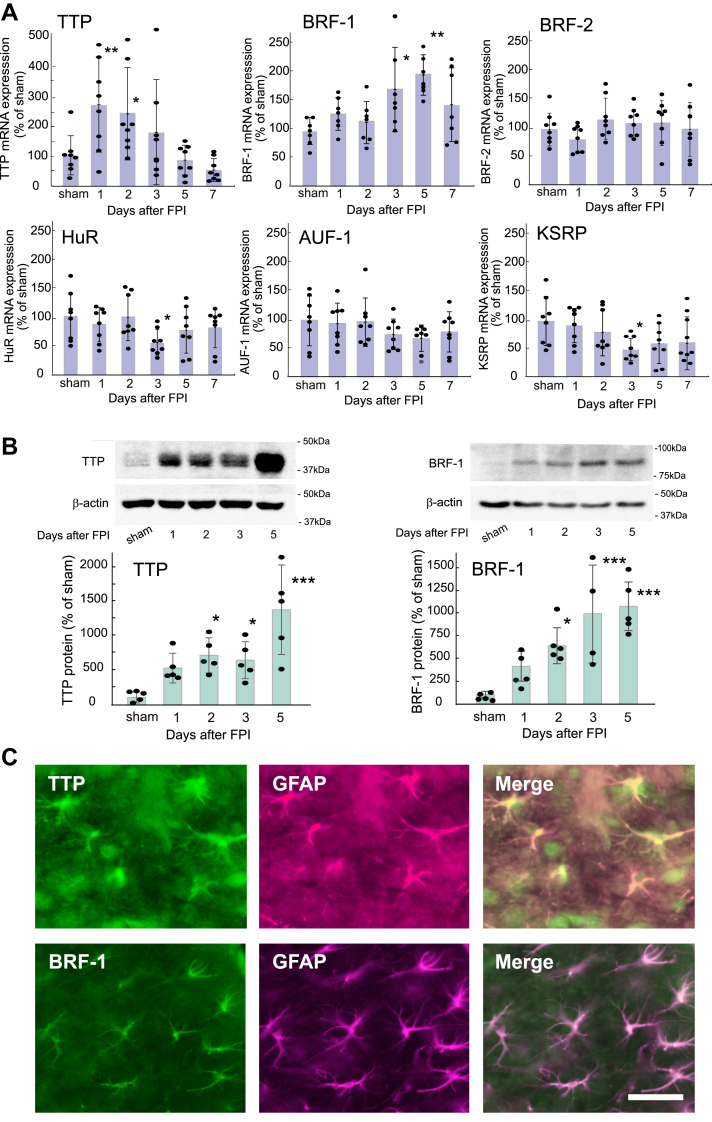
**Effects of FPI on RBP expression levels in the mouse cerebrum**. *A*, TTP and BRF mRNA expression levels after FPI. At 1 − 7 days after FPI, 5-mm-thick coronal brain slices containing the injured area were prepared, and the cerebrum was dissected from the slice. The expression levels of RBP mRNA in the cerebrum were measured by quantitative RT-PCR. The results were presented as mean ± SD of the findings for seven to eight mice. Shapiro–Wilk test showed that the data for all groups were normally distributed (*p* = 0.1681–0.9723). Therefore, one-way ANOVA was applied [TTP;*F* (5,42) = 3.273, *p* = 0.0138, BRF-1;*F* (5,42) = 2.972, *p* = 0.0164]. ∗*p* < 0.05, ∗∗*p* < 0.01 *versus* sham (FPI 0 days) group by Dunnett test. *B*, TTP and BRF-1 protein expression after FPI. At 1 − 5 days after FPI, TTP and BRF-1 expression levels in the cerebrum were measured by immunoblot analysis. The results are presented as mean ± SD of the findings for four to five mice. Shapiro–Wilk test showed that the data for all groups were normally distributed (*p* = 0.0590–0.9345). Therefore, one-way ANOVA was applied [TTP;*F* (4,20) = 8.565, *p* = 3.383 × 10^-4^, BRF-1;*F* (4,20) = 8.215, *p* = 5.051 × 10^-4^]. ∗*p* < 0.05, ∗∗∗*p* < 0.001 *versus* sham group by Dunnett test. *C*, Immunohistochemical observation of TTP and BRF-1 in the mouse cerebrum. Two days after FPI, TTP and BRF-1 expression in the injured area of mouse brain was observed by a double-labeling with anti-GFAP antibody. Scale bar = 50 μm.

**Figure 4:**
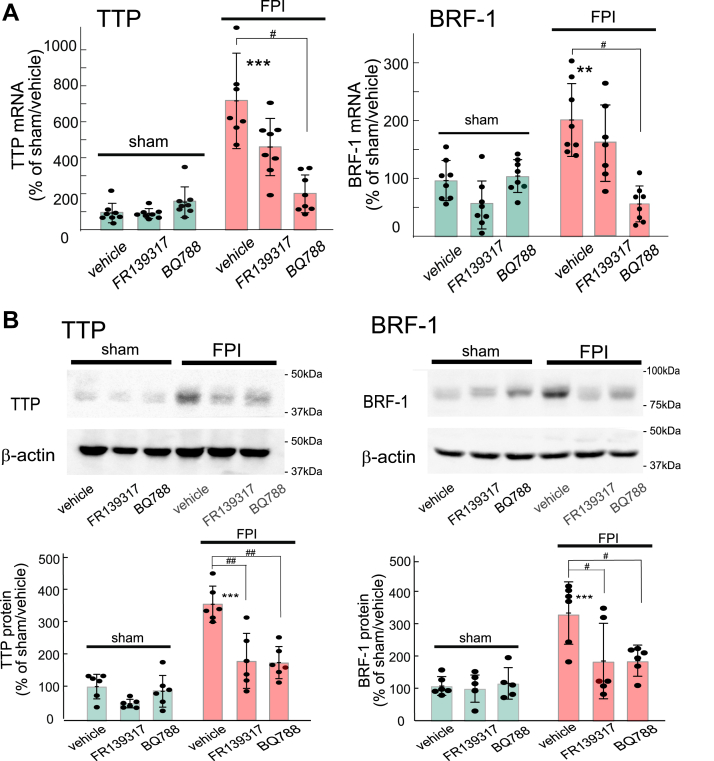
**Effects of ET antagonists on FPI-induced increments in TTP and BRF-1 expression**. *A*, mRNA. For measurement of TTP, FR139317 (15 nmol/day) and BQ788 (15 nmol/day) were repeatedly administered through the intracerebroventricular route 30 min and 24 h after FPI. In the control group, sterilized saline containing 1% dimethyl sulfoxide was administered as a vehicle. Two days after FPI, the injured cerebrum (*left side*) was removed and total RNA was prepared. For measurement of BRF-1, FR139317 (15 nmol/day) and BQ788 (15 nmol/day) were repeatedly administered 3 and 4 days after FPI. Five days after FPI, total RNA was prepared from the cerebrum. Results are presented as mean ± SD of the findings from seven to eight mice. Shapiro–Wilk test showed that the data for all groups were normally distributed (*p* = 0.0891–0.9142). Therefore, one-way ANOVA was applied [TTP;*F* (5,39) = 18.95, *p* = 1.596 × 10^-9^, BRF-1;*F* (5,41) = 12.35, *p* = 2.438 × 10^-7^]. ∗∗*p* < 0.01, ∗∗∗*p* < 0.001 *versus* sham/vehicle, ^#^*p* < 0.05 *versus* FPI/vehicle by Tukey test. *B*, protein. For measurement of TTP, FR139317 (15 nmol/day) and BQ788 (15 nmol/day) were repeatedly administered through the intracerebroventricular route 30 min and 24 h after FPI. Two days after FPI, the cerebrum was removed and tissue lysate was prepared. For measurement of BRF-1, FR139317 (15 nmol/day) and BQ788 (15 nmol/day) were repeatedly administered 3 and 4 days after FPI. Five days after FPI, tissue lysate was prepared from the cerebrum. Results are presented as mean ± SD of the results from five to six mice. Shapiro–Wilk test showed that the data for all groups were normally distributed (*p* = 0.0882–0.8289). Therefore, one-way ANOVA was applied [TTP;*F* (5,30) = 26.81, *p* = 3.168 × 10^-10^, BRF-1;*F* (5,28) = 11.26, *p* = 5.073 × 10^-6^]. ∗∗∗*p* < 0.001 *versus* sham/vehicle, ^#^*p* < 0.05, ^##^*p* < 0.01 *versus* FPI/vehicle by Tukey test.

FPI to the mouse brain increased TTP mRNA levels in the cerebrum 1 to 2 days after injury; however, the increased mRNA levels declined after 3 to 7 days (Fig. 3*A*). FPI also increased BRF-1 mRNA levels after 3 to 5 days (Fig. 3*A*). The TTP and BRF-1 protein levels were increased 2 to 5 days after FPI (Fig. 3*B*). The expression levels of HuR and KSRP mRNA were reduced only 3 days after FPI, while the expression levels of BRF-2 and AUF-1 were not affected by FPI (Fig. 3*A*). Immunohistochemical analysis revealed that FPI to the mouse cerebrum increased the number of glial fibrillary acidic protein (GFAP)-positive reactive astrocytes, which were observed in small numbers in the uninjured (sham) mice(Fig. S2). Cells expressing TTP or BRF-1 were scant in the uninjured mice, but these proteins were expressed in reactive astrocytes in the injured mouse cerebrum (Figs. 3*C* and S2). We confirmed that negative control staining, in which anti-TTP and BRF-1 antibodies were replaced with normal rabbit and mouse IgG, respectively, did not show any colocalization with GFAP-positive cells (Fig. S3). The FPI-induced increase in TTP and BRF-1 mRNA expression levels was reduced by intracerebroventricular administration of, 15 nmol BQ788 (Fig. 4*A*). Although FR137319 (15 nmol) also decreased the effects of FPI, these effects were not statistically significant. Intracerebroventricular administration of BQ788 reduced the FPI-induced increments in the expression of TTP and BRF-1 proteins (Fig. 4*B*). In contrast to the results of mRNA measurements, FPI-induced increments in TTP and BRF-1 protein levels were reduced by intracerebroventricular administration of FR19317 to a similar extent as BQ788 (Fig. 4*B*).

### Induction of astrocytic chemokines/cytokines by ET-1

**Figure 5:**
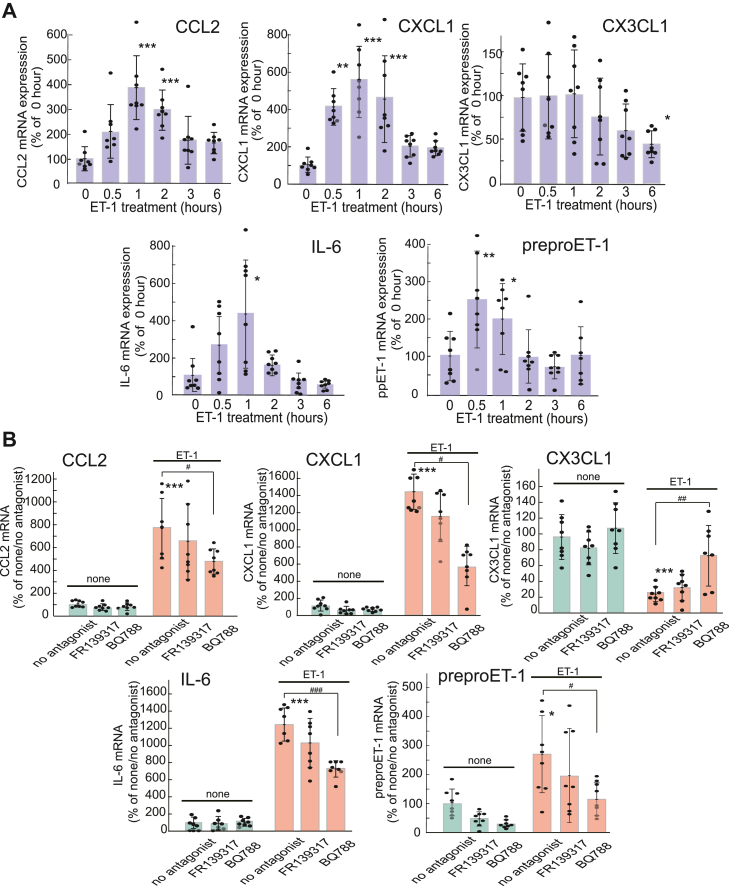
**Effects of ET-1 on astrocytic cytokine/chemokine expression**. *A*, cultured astrocytes were treated with 100 nM ET-1 for the durations indicated. The expression levels of cytokine/chemokine mRNAs were normalized to that of G3PDH. The results are presented as the mean ± SD of the findings for seven to eight different mRNA preparations. Shapiro–Wilk test showed that the data for all groups were normally distributed (*p* = 0.0914–0.9999). Therefore, one-way ANOVA was applied [CCL2;*F* (5,42) = 9.806, *p* = 2.914 × 10^-6^, CXCL1;*F* (5,42) = 8.623, *p* = 1.098 × 10^-5^. CX3CL1;*F* (5,42) = 18.66, *p* = 0.0267, IL-6;*F* (5,36) = 3.469, *p* = 0.0116, preproET-1;*F* (5,41) = 5.936, *p* = 3.230 × 10^-4^]. ∗*p* < 0.05, ∗∗*p* < 0.01, ∗∗∗*p* < 0.001 *versus* no ET-1 treatment (0 h) by o Dunnett test. *B*, effects of FR139317 and BQ788 on ET-induced cytokine/chemokine expression. Cultured astrocytes were treated with 100 nM ET-1 for 1 h in the presence or absence of 1 μM BQ788 and 1 μM FR139317. The results are presented as mean ± SD of the findings for seven to eight different mRNA preparations. Shapiro–Wilk test showed that the data for all groups were normally distributed (*p* = 0.0689–0.9720). Therefore, one-way ANOVA was applied [CCL2;*F* (5,42) = 23.87, *p* = 2.729 × 10^-11^, CXCL1;*F* (5,42) = 48.64, *p* = 2.127 × 10^-16^. CX3CL1;*F* (5,40) = 15.91, *p* = 1.276 × 10^-8^, IL-6;*F* (5,42) = 64.02, *p* = 1.490 × 10^-18^, preproET-1;*F* (5,41) = 6.492, *p* = 1.568 × 10^-4^]. ∗∗*p* < 0.01, ∗∗∗*p* < 0.001 *versus* none/no antagonist, ^#^*p* < 0.05, ^###^*p* < 0.001 *versus* ET-1/no antagonist by Tukey test.

In nerve tissue that has undergone TBI, reactive astrocytes release various cytokines and chemokines (8). Therefore, we examined the effects of ET-1 on the production of astrocytic cytokines and chemokines. Treatment of cultured astrocytes with 100 nM ET-1 caused a transient increase in CCL2, CXCL1, IL-6, and preproET-1 mRNA levels at 1 to 2 h, whereas the increased mRNA levels returned to baseline within 6 h (Fig. 5*A*). The expression of CX3CL1 did not increase but was decreased by ET-1. The expression levels of CCL5, CXCL12, IL-1β, and tumor necrosis factor-α (TNFα) mRNAs were not affected by ET-1 (Fig. S4). The effects of ET-1 on astrocytic CCL2, CXCL1, IL-6, preproET-1, and CX3CL1 expression were reduced by BQ788 but not by FR139317(Fig. 5*B*).

**Figure 6:**
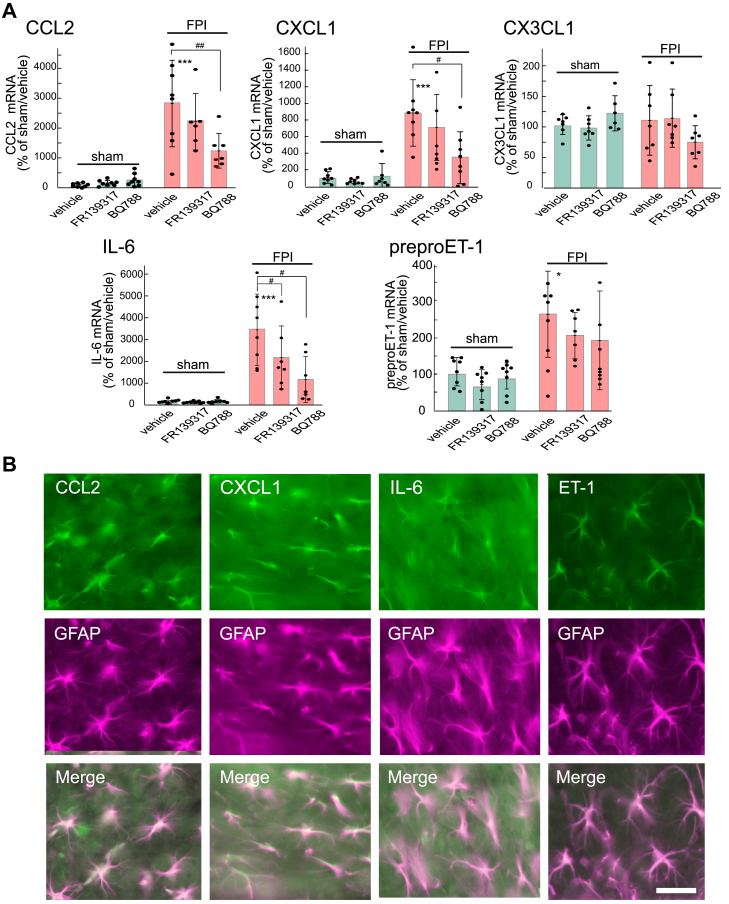
**Cytokine/chemokine expression in the mouse TBI model**. *A*, Effects of intracerebroventricular administration of ET antagonists on the FPI-induced increments in cytokine/chemokine expression. FR139317 (15 nmol/day) and BQ788 (15 nmol/day) were administered 30 min and 24 h after FPI. In the control group, sterilized saline containing 1% dimethyl sulfoxide was administered as the vehicle. Two days after FPI, the injured cerebrum was removed, and the amounts of cytokine/chemokine mRNA were measured. Results are presented as mean ± SD of the findings for six to eight mice. Shapiro–Wilk test showed that the data for all groups were normally distributed (*p* = 0.0745–0.8964). Therefore, one-way ANOVA was applied [CCL2;*F* (5,39) = 17.51, *p* = 4.529 × 10^-9^, CXCL1;*F* (5,40) = 9.331, *p* = 5.961 × 10^-6^. IL-6;*F* (5,40) = 9.637, *p* = 4.285 × 10^-6^, preproET-1;*F* (5,41) = 6.397, *p* = 1.774 × 10^-4^]. ∗*p* < 0.05, ∗∗∗*p* < 0.001 *versus* sham/vehicle, ^#^*p* < 0.05, ^##^*p* < 0.01 *versus* FPI/vehicle by Tukey test. *B*, Immunohistochemical analyses of CCL2, CXCL1, IL-6, and ET-1 expression in the mouse cerebrum. Two days after FPI, the expression of CCL2, CXCL1, IL-6, and ET-1 in the injured area of the mouse brain was observed by double-labeling with anti-GFAP antibody. Scale bar = 50 μm.

The mRNA expression levels of CCL2, CXCL1, IL-6, and preproET-1 in the injured cerebrum increased 2 days after FPI (Fig. 6*A*). Immunohistochemical observations showed that CCL2, CXCL1, IL-6, and ET-1 were expressed in GFAP-positive astrocytes in the cerebrum 2 days after FPI (Fig. 6*B*). The FPI-induced increments in CCL2, CXCL1, and IL-6 expression were reduced by the administration of BQ788 (15 nmol) (Fig. 6*A*). The FPI-induced expression of preproET-1 was not affected by BQ788. FR139317 (15 nmol) reduced FPI-induced IL-6 expression but had no effect on the expression of CCL2, CXCL1, IL-6, and preproET-1. CX3CL1 expression was not affected by FPI or administration of FR139317 or BQ788.

### Regulation of CCL2 and IL-6 expression by TTP

**Figure 7:**
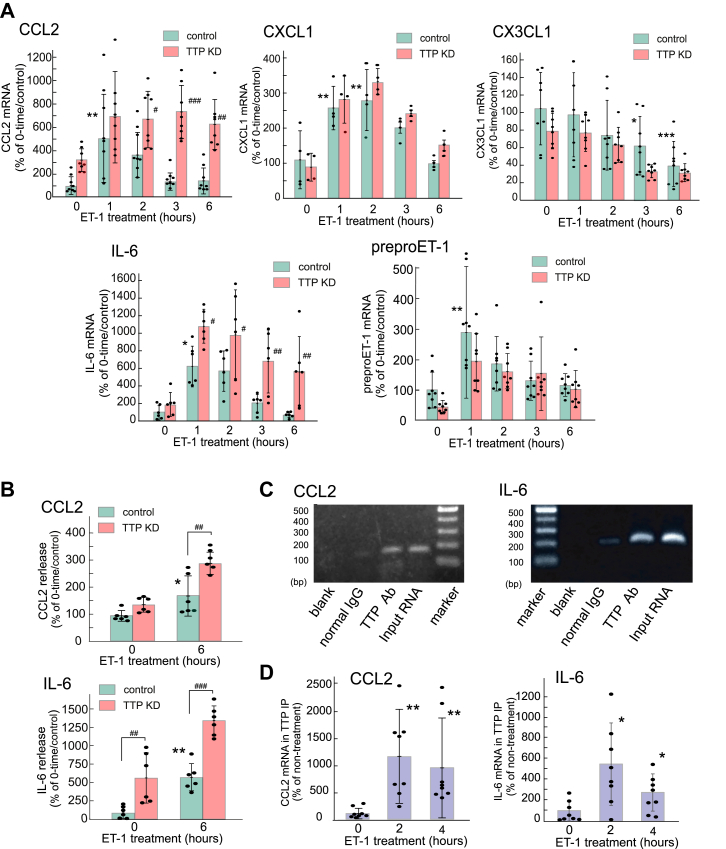
**Effect of TTP siRNA on the ET-induced increase in cytokine/chemokine expression**. *A*, mRNA expression. Cultured astrocytes were transfected with TTP siRNA or scrambled RNA, as described in the Materials and Methods. Total RNA was prepared after the transfected astrocytes were treated with 100 nM ET-1 for 1 to 6 h. The results are presented as mean ± SD of the findings for four to eight different RNA preparations. Shapiro–Wilk test showed that the data for all groups were normally distributed (*p* = 0.0666–0.9358). Therefore, the effect of TTP siRNA was analyzed by two-way ANOVA [CCL2;*F* (1,70) = 30.16, *p* = 6.014 × 10^-7^, IL-6;*F* (1,50) = 10.77, *p* = 1.878 × 10^-3^]. *∗p* < 0.05, *∗∗p* < 0.01, *∗∗∗p* < 0.001 *versus* control/no ET-1 treatment (0 h), ^#^*p* < 0.05, ^##^*p* < 0.01, ^###^*p* < 0.001 *versus* each control by Tukey’s test. *B*, CCL2 and IL-6 release. TTP siRNA-transfected astrocytes were treated with 100 nM ET-1 in serum-free MEM for 6 h, and cultured medium was collected subsequently. The amounts of CCL2 and IL-6 in the cultured medium were measured by ELISA. Results are presented as mean ± SD of the findings for six different preparations. Shapiro–Wilk test showed that the data for all groups were normally distributed (*p* = 0.1264–0.9544). Therefore, one-way ANOVA was applied [CCL2;*F* (3,20) = 25.57, *p* = 4.757 × 10^-7^, IL-6;*F* (3,20) = 40.84, *p* = 1.023 × 10^-8^]. ∗*p* < 0.05, ∗∗*p* < 0.01 *versus* control/no ET-1 treatment (0 h), ^#^*p* < 0.05, ^##^*p* < 0.01, ^###^*p* < 0.001 *versus* each control by Tukey’s test. *C*, association of the TTP protein with the 3′-UTRs of CCL2 and IL-6 mRNAs. The cell lysate of paraformaldehyde-fixed cultured astrocytes was subjected to RNA/IP with an anti-TTP mouse antibody. Non-immune mouse IgG was included in the RNA/IP mixture as the negative control. Total RNA was extracted from the immunoprecipitants and cDNA was prepared. cDNA was amplified by PCR using the primer pair indicated in Figure S6. cDNA directly prepared from total RNA was similarly amplified as input DNA, and sterile H_2_O was used as the blank. The PCR products were electrophoresed in 2% agarose gel and stained with 1 μg/ml ethidium bromide. Unprocessed gel images are presented in the Supporting information. *D*, Effects of ET-1 on TTP binding to 3′-UTRs of CCL-2 and IL-6 mRNAs. After cultured astrocytes were treated with 100 nM ET-1 for 2 and 4 h, the cell lysate was prepared. After RIP using an anti-TTP antibody, total RNA was extracted from the immunoprecipitants. 3′-UTRs of CCL2 and IL-6 mRNAs binding to TTP were measured by quantitative PCR. The results are presented as mean ± SD of the findings for eight different cell lysates. Shapiro–Wilk test showed non-normally distributed groups (*p* = 0.0013–0.9054). Therefore, Kruskal-Wallis test was applied [CCL2; *H*(2) = 13.90, *p* = 9.562 × 10^-4^, IL-6; *H*(2) = 8.795, *p* = 0.0123]. ∗*p* < 0.05, ∗∗*p* < 0.01 *versus* no ET-1 treatment (0 h) by Steel-Dwass test.

To demonstrate the involvement of TTP in the astrocytic chemokine/cytokine production induced by ET-1, the effect of TTP knockdown using siRNA was examined. Transfection of cultured astrocytes with TTP siRNA reduced the TTP mRNA levels to approximately 30% of the negative control in the non-ET-1 treatment group (Fig. S5). ET-induced astrocytic TTP mRNA expression was also largely reduced by TTP knockdown. As shown in Figure 5*A*, treatment with ET-1 for 1 h transiently increased CCL2 and IL-6 mRNA levels in control astrocytes, and these increments gradually declined. TTP siRNA potentiated the ET-induced expression of astrocytic CCL2 and IL-6 mRNA (Fig. 7*A*). The release of CCL2 and IL-6 from cultured astrocytes was stimulated by ET-1, and the effects of ET-1 were also potentiated by TTP siRNA (Fig. 7*B*). Transfection with TTP siRNA did not affect ET-induced alterations in CXCL1, CX3CL1, and preproET-1 mRNA expression (Fig. 7*A*). We examined whether TTP regulates CCL2 and IL-6 expression by directly binding to mRNA using RNA immunoprecipitation (RIP)/PCR with an anti-TTP antibody. Each PCR primer pair was designed to amplify the 3′-UTR-containing AREs of rat CCL2 and IL-6 (Fig. S6). These primer pairs were confirmed to amplify the 3′-UTRs of CCL2 and IL-6 mRNAs in the TTP immunoprecipitant (Fig. 7*C*). The 3′-UTRs of CCL2 and IL-6 mRNAs in the TTP immunoprecipitant were increased by treatment with 100 nM ET-1 (Fig. 7*D*).

### Regulation of CCL2, CXCL1, and CX3CL1 expression by BRF-1

**Figure 8:**
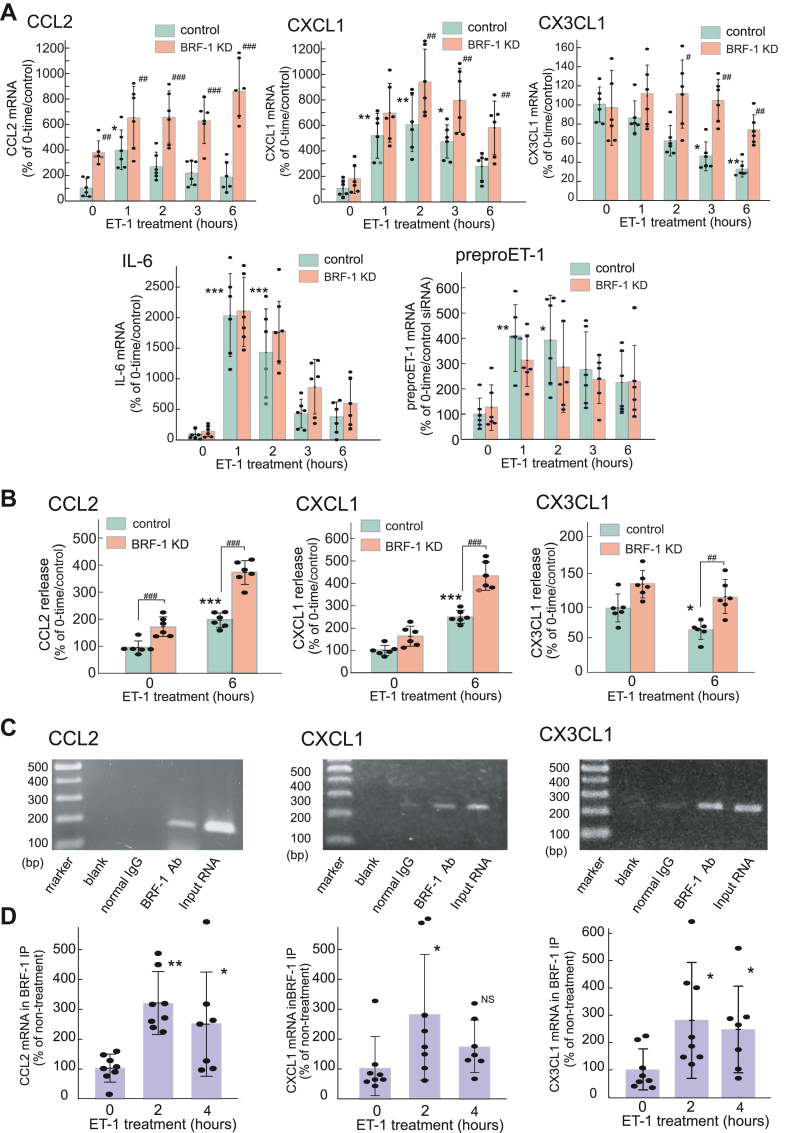
**Effect of BRF-1 siRNA on the ET-induced increase in cytokine/chemokine expression**. *A*, mRNA. Cultured astrocytes were transfected with BRF-1 siRNA or scrambled RNA. Total RNA was prepared after treatment with ET-1 for 1 to 6 h. The results are presented as mean ± SD of the findings for six to eight different RNA preparations. Shapiro–Wilk test showed that the data for all groups were normally distributed (*p* = 0.0856–0.9516). Therefore, the effect of BRF-1 siRNA was analyzed by two-way ANOVA [CCL2;*F* (1,50) = 98.20, *p* = 2.180 × 10^-13^, CXCL1;*F* (1,50) = 26.22, *p* = 4.880 × 10^-6^, CX3CL1;*F* (1,50) = 27.32, *p* = 3.380 × 10^-6^]. ∗*p* < 0.05, ∗∗*p* < 0.01, *∗∗∗p* < 0.001 *versus* control/no ET-1 treatment (0 h), ^#^*p* < 0.05, ^##^*p* < 0.01, ^###^*p* < 0.001 *versus* each control by Tukey’s test. *B*, effects on the release of CCL2, CXCL1, and CX3CL1. BRF-1 siRNA-transfected astrocytes were treated with 100 nM ET-1 for 6 h. The amounts of CCL2, CXCL1, and CX3CL1 in the cultured medium were measured by ELISA. The results are presented as mean ± SD of the findings for six different preparations. Shapiro–Wilk test showed that the data for all groups were normally distributed (*p* = 0.2855–0.9182). Therefore, one-way ANOVA was applied [CCL2;*F* (3,20) = 58.29, *p* = 4.566 x 10^-10^, CXCL1;*F* (3,20) = 69.48, *p* = 9.388 x10^-11^, CX3CL1;*F* (3,20) = 12.92, *p* = 6.389 x10^-5^]. *∗p* < 0.05, *∗∗∗p* < 0.001 *versus* no ET-1 treatment (0 h), ^##^*p* < 0.01, ^###^*p* < 0.001 *versus* each control by Tukey test. *C*, association of BRF-1 protein with the 3′-UTRs of CCL2, CXCL1, and CX3CL1 mRNAs. Astrocytic lysate was subjected to RNA/IP with an anti-BRF-1 mouse antibody. Non-immune mouse IgG was used as the negative control. Total RNA was extracted from the immunoprecipitants and cDNA was prepared. The cDNA was amplified by PCR using the primer pair indicated in Figure S6. cDNA directly prepared from the total RNA was similarly amplified as input DNA, and sterile H_2_O was used as blank. Unprocessed gel images are presented in the Supporting information. *D*, effects of ET-1 on BRF-1 binding to the 3′-UTRs of CCL2, CXCL1, and CX3CL1 mRNAs. Cultured astrocytes were treated with 100 nM ET-1 for 2 and 4 h. After RIP using an anti-BRF-1 antibody, total RNA was extracted from the immunoprecipitants. 3′-UTRs of CCL2, CXCL1, and CX3CL1 mRNAs were measured by quantitative PCR. The results are presented as mean ± SD of the findings for five to seven to eight different cell lysates. Shapiro–Wilk test showed non-normally distributed groups (*p* = 0.0015–0.5705). Therefore, Kruskal-Wallis test was applied [CCL2; *H*(2) = 10.60, *p* = 0.0049, CXCL1; *H*(2) = 6.046, *p* = 0.0486, CX3CL1; *H*(2) = 6.441, *p* = 0.0399]. ∗*p* < 0.05, ∗∗*p* < 0.01 *versus* no ET-1 treatment (0 h) by Steel-Dwass test.

Transfection of cultured astrocytes with BRF-1 siRNA reduced BRF-1 mRNA levels to approximately 35% of the negative control in the non-ET-1 treatment group (Fig. S5). ET-induced astrocytic BRF-1 mRNA expression was also largely reduced by BRF-1 knockdown. Treatment with ET-1 for 1 to 2 h increased the levels of CCL2 and CXCL1 mRNAs in control astrocytes (Fig. 8*A*). BRF-1 siRNA potentiated ET-induced astrocytic CCL2 and CXCL1 mRNA expression. Although ET-1 decreased CX3CL1 mRNA expression in control astrocytes, the effect of ET-1 was not observed with BRF-1 knockdown. ET-1 stimulated the release of CCL2 and CXCL1 from cultured astrocytes, and the effects of ET-1 were potentiated by BRF-1 siRNA (Fig. 8*B*). ET-1 decreased CX3CL1 release from cultured astrocytes, and this effect of ET-1 was reduced by BRF-1 knockdown. BRF-1 knockdown did not affect the ET-induced increments in IL-6 and preproET-1 mRNA expression (Fig. 8*A*). RIP/PCR using an anti-BRF-1 antibody showed that the BRF-1 immunoprecipitant contained the 3′-UTRs of rat CCL2, CXCL1 and CX3CL1 mRNAs (Fig. 8*C*). The 3′-UTRs of CCL2, CXCL1, and CX3CL1 mRNAs in the BRF-1 immunoprecipitant were increased by treatment with 100 nM ET-1 (Fig. 8*D*).

## Discussion

### ET-1 increases the expression of TTP and BRF-1 in astrocytes through ETB receptors

In brain disorders, including TBI, astrocytes transition to the reactive phenotype. Reactive astrocytes produce various chemokines and cytokines that exacerbate neuroinflammation (3, 4, 5, 6, 7, 8). Studies on immune cells have indicated the pivotal roles of Zfp36 family RBPs in regulating the expression of some proinflammatory factors (13, 14). As for TTP, a few reports have shown increased expression of TTP in brain ischemia models (19, 20) and cultured astrocytes (21). However, the regulation of astrocytic Zfp36 family expression in TBI and their roles in the production of astrocytic cytokines/chemokines are largely unknown. In the present study, ET-1 stimulated TTP and BRF-1 expression through ET_B_ receptors in cultured astrocytes (Fig. 1 and 2). FPI on the mouse cerebrum increased the expression of TTP and BRF-1 in astrocytes (Fig. 3), and these effects were reduced by BQ788 (Fig. 4). These results clearly indicate that the activation of ET_B_ receptors induces TTP and BRF-1 expression in astrocytes. In the brain damage caused by TBI, the production of ET-1 increased and promoted the induction of reactive astrocytes (29, 30). Therefore, ET-1 may be a major signaling molecule that induces astrocytic TTP and BRF-1 expression in TBI.

On the other hand, the contribution of ET_A_ receptors to the increased TTP and BRF-1 in a TBI model was suggested to be different from that of ET_B_ receptors. That is, FR139317 reduced the protein levels of TTP and BRF-1 to the same extent as BQ788 (Fig. 4*B*) but did not affect TTP and BRF-1 mRNA expression in cultured astrocytes (Fig. 2*B*). While these results show an involvement of ET_A_ receptors expressed on cells other than astrocytes, the present study did not further examine the mechanism mediated by ET_A_ receptors in the TBI model. In the brain, ET_A_ receptors are expressed on cerebral blood vessels, and their activation exacerbates ischemic injury through vasospasm (22, 31, 32, 33). So it is possible that exacerbation of brain ischemia by activation of ET_A_ receptors increases TTP and BRF-1 expression in the TBI model.

### Regulation of astrocytic chemokine/cytokine expression by ETB receptors

Some of proinflammatory factors produced by activated astrocytes have ARE sequences in the 3′-UTR of mRNA. To identify the target molecules of astrocytic TTP and BRF-1, we examined the effects of ET-1 on chemokine and cytokine mRNAs with ARE sequences (Fig. 5). In this study, the mRNA expression levels of CCL2, CXCL1, IL-6, and preproET-1 rapidly decreased following transient upregulation (Fig. 5*A*). In the present study, increased expression levels of CCL2, CXCL1, IL-6, and preproET-1 in activated astrocytes was observed in our FPI-induced TBI model (Fig. 6). Moreover, for CCL2, CXCL1 and IL-6, BQ788 reduced the FPI-induced increments in mRNA levels (Fig. 6*A*). Although many extracellular signals are involved in proinflammatory factor production in brain disorders, the effects of the ET_B_ antagonist suggest that the increments in CCL2, CXCL1, and IL-6 production in TBI are largely mediated by the activation of astrocytic ET_B_ receptors. In contrast, the effects of BQ788 on preproET-1 and CX3CL1 expression differed between cultured astrocytes and TBI models (Figs. 5*B* and 6*A*). In the brain, ET-1 and CX3CL1 are not only produced by astrocytes but also by neurons and vascular endothelial cells (34, 35, 36). No inhibitory effect of BQ788 on TBI-induced ET-1 and CX3CL1 production (Fig. 6*A*) may suggest a low contribution of astrocytes as a source of these substances.

### Increased expression of TTP and BRF-1 negatively regulate the production of CCL2, CXCL1, CX3CL1, and IL-6

In the present study, TTP and BRF-1 were found to be involved in the regulation of astrocyte chemokines and cytokines. Knockdown of TTP enhanced the ET-1-induced increments in CCL2 and IL-6 expression (Fig. 7). BRF-1 knockdown also enhanced the expression of CCL2, CXCL1, and CX3CL1 (Fig. 8). These results suggest that an increase in TTP production negatively regulates astrocytic CCL2 and IL-6 expression, whereas that in BRF-1 negatively regulates CCL2, CXCL1, and CX3CL1 expression. Zfp36 family proteins regulate the expression of cytokine/chemokine productions through binding to the 3′-UTR of target mRNAs (13, 14). The RIP/PCR experiments using a TTP antibody showed that TTP protein bound to the 3′-UTR of CCL2 and IL-6 mRNAs, and that the binding activity was stimulated by ET-1 (Fig. 7C). In addition, the BRF-1 protein bound to the 3′-UTR of CCL2, CXCL1, and CX3CL1 mRNAs, and the binding was stimulated by ET-1 (Fig. 8*C*). These results indicate that TTP and BRF-1 reduce the expression of astrocytic proinflammatory factors through post-transcriptional destabilization of their mRNAs. However, previous studies on other cell types have shown the negative regulation of CXCL1 by TTP (37, 38, 39) and IL-6 by BRF-1 (40), which differ from the present findings in astrocytes. The present study did not clarify the mechanisms underlying the differences in the target cytokine/chemokine mRNAs of TTP and BRF-1 between cell types. Zfp36 family RBPs have been reported to reduce the expression of proinflammatory factors not only by mRNA degradation, but also by suppressing the activity of nuclear factor-ƙB (41, 42). In addition, Reznik *et al*. (43) proposed the existence of a cofactor that binds to TTP and BRF1 to regulate mRNA degradation activity. Differences in the transcriptional regulation and involvement of cofactors between cell types may determine the preference of target cytokine/chemokine mRNAs of TTP and BRF-1.

The present observations in cultured cells show that increased TTP and BRF-1 in astrocytes negatively regulate cytokine/chemokine production. Furthermore, increased astrocytic TTP and BRF-1 production was observed in a TBI model. However, the present results will be insufficient to clarify what extent the negative regulation by ET-induced astrocytic TTP and BRF-1 affects cytokine/chemokine production in TBI. It is because microglia and inflammatory blood cells also produce cytokines/chemokines in TBI. To clarify this issue, it will be necessary to examine the effects of astrocyte-specific TTP or BRF-1 knockdown in the TBI animal model.

### Possible significance of TTP and BRF-1 on the regulation of astrocytic cytokine/chemokine production

**Figure 9:**
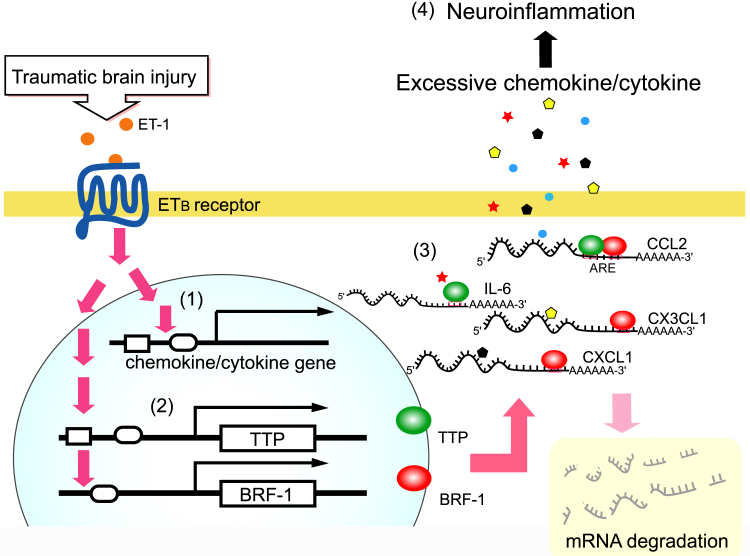
**Possible roles of astrocytic TTP and BRF-1 in ET-induced cytokine/chemokine productions**. On TBI, (1) brain ET-1 is increased and stimulates cytokine/chemokine production from astrocytes, which is involved in the induction of brain edema and neuroinflammation. (2) At the same time, ET-1 increases production of TTP and BRF-1. (3) TTP and BRF-1 bind to astrocytic cytokine/chemokine mRNAs and promote their degradation. (4) As a result, cytokine/chemokine productions by astrocytes are reduced.

The present study clearly showed that activation of astrocytic ET_B_ receptors stimulated CCL2, CXCL1 and IL-6 production, and triggered a negative regulatory mechanism in cytokine/chemokine production through TTP and BRF-1 (Figs. 7 and 8). In the brain, astrocytes surround brain microvessels with their endfeet and increase the permeability of microvessels by releasing various vascular permeability regulatory factors, including CCL2, CXCL1, and IL-6. Astrocyte-derived CCL2, CXCL1, and IL-6 are excessively produced in TBI (5, 6, 7, 8), inducing BBB disruption and brain edema (44, 45, 46). In patients and animal models of TBI, the production of brain ET-1 increases (30, 47, 48, 49) and induces pathophysiological responses in astrocytes through activation of ET_B_ receptors. We had previously observed that administration of BQ788 alleviated BBB disruption and brain edema in a mouse model of TBI (22, 30). Therefore, the reduction in TBI-induced CCL2, CXCL1, and IL-6 production by BQ788 (Fig. 6) suggest that reduced cytokine/chemokine production underlies the protective effect of BQ788 against BBB disruption and brain edema. On the other hand, as for the roles of Zfp36 family proteins in brain edema, Li *et al*. (19) reported that TTP suppressed brain edema in a cerebral hemorrhage model. Increased BBB permeability in brain disorders is essentially considered to play a role in promoting the regeneration of neural tissue by allowing the infiltration of inflammatory blood cells to remove cell debris in the brain. However, excessive increases in BBB permeability can induce brain edema and neuroinflammation, resulting in exacerbation of brain damage. Therefore, the negative regulation of cytokine/chemokine production by TTP and BRF-1 may be an intrinsic cellular mechanism by which astrocytes prevent brain edema and neuroinflammation (Fig. 9).

## Conclusions

The activation of ET_B_ receptors in astrocytes stimulated the expression of TTP and BRF-1 in cultured cells and TBI models. Increased expression of TTP and BRF-1 negatively regulated the expression of astrocytic CCL2, CXCL1, CX3CL1, and IL-6. These findings indicate that a negative regulatory mechanism downstream of astrocytic ET_B_ receptors is triggered to prevent excess production of proinflammatory factors by ET-1.

## Experimental procedures

### Experimental animals

All animal experimental protocols were performed in accordance with the National Institutes of Health (NIH) guidelines for the care and use of animals (NIH Publication No. 8023) and approved by the Animal Experiment Committee of Kobe Pharmaceutical University (Approval No. 2025–043) and Meiji Pharmaceutical University (Approval No. 202206). All animals were housed under pathogen-free conditions and provided a commercial diet and water ad libitum under controlled conditions (temperature: 23 ± 2 °C, humidity: 55% ± 5%) with a 12-h light/dark cycle.

### Preparation of primary cultured astrocytes from the rat brain

Astrocytes were prepared from the cerebra of 1- to 2-day-old Wistar rats (Japan SLC, Inc), as described previously (50). Isolated cells were seeded at 1 × 10^4^ cells/cm^2^ in 75-cm^2^ culture flasks and grown in minimum essential medium (MEM) supplemented with 10% fetal calf serum. To remove small process-bearing cells (mainly oligodendrocyte progenitors and microglia) from the protoplasmic cell layer, the culture flasks were shaken at 250 rpm overnight for 10 to 14 days after seeding. Monolayer cells were trypsinized and seeded into 6-well culture plates. At this stage, approximately 92% of cells showed immunoreactivity for aldehyde dehydrogenase one family member L1 (ALDH1L1). Primary cultured neurons and microglia from rat cerebrum were prepared as described previously (50).

### Fluid percussion injury to the mouse cerebrum

For an animal model of TBI, 6–7-week-old male ddy mice (Japan SLC, Inc) were used. Lateral fluid percussion injury (FPI) was induced using a fluid percussion device (model FP302; AmScien Instruments) in the mouse dura mater according to the protocol described in our previous study (29). To maintain anesthesia during induction of FPI, 0.3 mg/kg of medetomidine, 4.0 mg/kg of midazolam, and 5.0 mg/kg of butorphanol were administered intraperitoneally. Under anesthesia, the mice were fixed in a stereotactic device. The scalp was incised at the midline, and the skull was exposed. A 3-mm-diameter hole was made in the skull of the left hemisphere, 2.5 mm posterior and 2.5 mm left lateral from the bregma. After opening the skull, a lure fitting (cat#VRS306; ISIS, Japan) was fixed to the opening area using glue gel. The lure fitting was filled with sterilized saline, and the fixed lure fitting was connected to a polyethylene tube of the fluid percussion device. A hydraulic insult was applied in the determined range of 1.2−1.4 atm. Mice subjected to FPI that deviated from this range were excluded from subsequent experiments. In the sham-operated mice, the lure fitting was fixed to the left hemisphere in the same manner without a hydraulic insult. FR139317 (an ET_A_ antagonist; Funakoshi Co., Ltd) and BQ788 (an ET_B_ antagonist; Phoenix Pharmaceuticals Inc) were intracerebroventricularly administered into the mouse brain. To perform intracerebroventricular administration, a guide cannula (CXG-X; Eicom) was fixed to the skull of the right hemisphere in a position 0.1 mm posterior and 1.0 mm right lateral to the bregma using dental cement 3 days before FPI, as in our previous studies (22). The mice were randomly divided into sham and FPI groups, and the FPI groups were further divided into vehicle or drug administration groups.

### Measurement of mRNA levels by quantitative reverse transcription polymerase chain reaction

Total RNA was extracted from cultured cells and the mouse cerebrum using a total RNA extraction kit (Favorgen Biotech, Ping-Tung, Taiwan). First-strand complementary DNA (cDNA) was synthesized from total RNA (1 μg) using a reverse transcription premix kit (ReverTra Ace qPCR RT Master Mix Toyobo, Tokyo, Japan) according to the supplier’s protocol. The mRNA levels in each sample were determined by quantitative polymerase chain reaction (PCR) using SYBR Green fluorescent probes. Each reverse transcription product was added to the SYBR Green Master Mix NEXT (Toyobo) along with the primer pairs, and the mixture was placed in a thermal cycler (CFX96 Connect; Bio-Rad). The sequences of the primer pairs used in this study are listed in Table S1. Serial dilutions of each amplicon were amplified in the same manner as the standard to determine the copy numbers of the PCR products. The amount of cDNA was calculated as the copy number of each reverse transcription product equivalent to 1 μg of total RNA and then normalized to the value of glyceraldehyde-3-phosphate dehydrogenase (G3PDH).

### Measurement of protein levels by immunoblotting

Cultured astrocytes in six-well culture plates were lysed in 100 μl of ice-cold lysis buffer (20 mM Tris/HCl, pH 7.4, 1% sodium dodecyl sulfate (SDS), 2 mM ethylenediaminetetraacetic acid, 2 mM phenylmethylsulfonyl fluoride, 20 μg/ml aprotinin) at 4 °C. To prepare mouse brain lysates, coronal brain sections (between 0 and 5 mm posterior to the bregma) were prepared, and the cerebrum was dissected from the brain sections. The cerebral tissue of each animal was homogenized in 200 μl of lysis buffer. The cell lysates were subjected to SDS polyacrylamide gel electrophoresis and electroblotted onto polyvinylidene fluoride membranes. For detection of TTP and BRF-1, the membranes were first probed with anti-TTP mouse antibody (cat# sc-374305; St Cruz Biotechnology) and anti-TFIIIB90 mouse antibody (cat# sc-390821; St Cruz Biotech). The membranes were then incubated with peroxidase-conjugated secondary antibodies. The labeled proteins were visualized using an enhanced chemiluminescence kit (Chemi-Lumi One L; Nacalai Tesque). The chemiluminescence of each band was recorded using an image analyzer (LuminoGraph II, Atto Corp), and the density of the target proteins was quantified using ImageJ 1.45 software (US NIH). After detection of TTP and BRF-1 proteins, the membranes were re-probed with mouse anti-β-actin primary antibody (cat#MAB1501; Chemicon). The expression levels of TTP and BRF-1 were indicated as a ratio to the expression level of β-actin protein. All immunoblots used for quantification are presented in the Supporting information.

### Determination of chemokines and cytokines released from cultured astrocytes

Cultured astrocytes in 6-well plates were treated with ET-1 in serum-free MEM, and the culture medium was collected. The amounts of chemokines/cytokines in the culture media were determined using the enzyme-linked immunosorbent assay (ELISA) kits for CCL2 (cat#BMS631INST; Invitrogen Rat MCP-1 Instant ELISA Kit, Fisher Scientific Inc.), CXCL1 (cat#KE20015; Proteintech Rat CXCL1 ELISA Kit, Fisher Scientifics Inc.), CX3CL1 (cat#ab100760; Rat Fractalkine ELISA Kit, Abcam), and IL-6 (cat# BMS625; Rat IL-6 ELISA Kit, Fisher Scientific Inc.) according to the manufacturers’ protocols. The protein content in each well was determined using a BCA protein assay kit (cat#FERA65453; Pierce BCA protein assay kits, Fisher Scientific Inc), and the results were normalized to the amount of chemokine/cytokine released per mg protein.

### Immunohistochemistry

Two days after TBI, mouse brains were intracardially perfused with 4% paraformaldehyde, and frozen brain sections were prepared as reported previously (29). Before incubation with the primary antibodies, frozen sections were autoclaved at 121 °C for 20 min in HistoVT ONE (cat#06380–76; Nacalai Tesque) for antigen retrieval. The brain sections were then incubated at 4 °C overnight with the primary antibodies listed in Table S2. To identify astrocytes, anti-GFAP rabbit (cat#12389; CST) or mouse (cat#MAB360; Sigma-Aldrich) antibody was included in the incubation with the primary antibodies. The labeled cells were then visualized with fluorescein isothiocyanate- or rhodamine-conjugated anti-IgG antibodies and observed under an epifluorescence microscope. As a negative control, brain sections were incubated with non-immune mouse (cat#sc-2025; St Cruz) or rabbit IgG (cat#30000-0-AP; Proteintech) instead of the primary antibodies.

### Knockdown of astrocytic TTP and BRF-1 by small interfering RNAs

Knockdown of astrocytic TTP and BRF-1 was achieved by transient transfection with small interfering RNA (siRNA). A mixture of three different siRNAs against rat TTP and BRF-1 was obtained from OriGene Tech Inc. (cat#SR515193-A, -B, and -C) and BioNeer Corp. (cat#313587–1, −2, and −3; Daejeon). The cultured astrocytes were transiently transfected with the mixture of siRNAs using Lipofectamine RNAiMAX (Thermo Fisher Scientific) according to the supplier’s protocol. Briefly, stock solutions of the siRNA and a negative control siRNA (cat#sc-3700;, St Cruz Biotech) were diluted to 200 nM in Opti MEM (Thermo Fisher Scientific). Meanwhile, Lipofectamine RNAiMAX was diluted to 2% in Opti MEM. The diluted siRNA and transfection reagent solutions were then mixed and incubated for 10 min to form an siRNA/transfection reagent complex. To examine the effects of the siRNAs on mRNA and protein expression, astrocytes were seeded into six-well plates. When cultured astrocytes reached 60% − 70% confluence, the siRNA and transfection reagent were added to serum-free MEM to final concentrations of 100 nM and 0.5%, respectively. Forty-eight hours later, the siRNA-containing medium was replaced with fresh serum-free MEM, and the cultured astrocytes were subjected to each experimental condition.

### RIP/PCR analysis of RBP binding to 3′-UTRs of chemokine/cytokine mRNAs

After incubation with 100 nM ET-1 for 2 and 4 h, cultured astrocytes in ϕ10 cm dishes were treated with paraformaldehyde (final concentration, 1.1%) to cross-link the RBPs and RNA. The fixed cells were washed with ice-cold phosphate-buffered saline and collected by centrifugation. Preparation of cell lysates, RIP, and extraction of RNA from immunoprecipitants were performed using an RIP-assay kit (cat#RN1001; RiboCluster Profiler, MBL, Tokyo, Japan) according to the supplier’s protocol. For immunoprecipitation of TTP-bound mRNA, cell lysate (50 mg protein) was incubated overnight at 4 °C with agarose-conjugated anti-TTP mouse antibody (IgG; 3 μg, cat# sc-374305AC; St Cruz Biotech, Beijing, China) using a rocking shaker. Equal amounts of agarose-conjugated normal mouse IgG (cat# sc-2343AC; St Cruz. Biotech) were used instead of the TTP antibody as the negative control. For BRF-1, cell lysates were incubated with anti-TFIIIB90 mouse antibody (3 μg, cat# sc-390821; St Cruz Biotech) and Protein A/G-agarose (cat# sc-2003; St Cruz Biotech). An equal amount of normal mouse IgG (cat# sc-2025; St Cruz Biotech) was used instead of the BRF-1 antibody as a negative control. mRNA extracted from the immunoprecipitants was reverse-transcribed using a reverse transcription premix kit (ReverTra Ace qPCR RT Master Mix, Toyobo). mRNA in the precipitate was quantified by reverse transcription PCR (RT-PCR) using a primer pair that amplified the 3′-UTR of each cytokine/chemokine mRNA (Table S1).

### Statistical analysis

Bell Curve for Excel (version 2.20, Social Survey Research Information Co. Ltd, Tokyo, Japan) was used for statistical analysis. Assessment of the normality of the data was performed by Shapiro–Wilk test, and the *p* value of 0.05 or greater was considered to be normally distributed. The data with a normal distribution were analyzed using one-way or two-way analysis of variance (ANOVA), followed by a *post hoc* test. The data with a non-normal distribution were analyzed using nonparametric analysis with Kruskal-Wallis test, followed by Steel-Dwass test. Statistical significance was set at *p* < 0.05. All experimental data are presented as the mean ± standard deviation (SD). Individual data points are indicated by dots.

## Data availability

The data supporting the findings of this study are available from the corresponding author upon reasonable request.

## Supporting information

This article contains supporting information.

## Conflict of interest

The authors declare that they do not have any conflicts of interest with the content of this article.
