# Supplementary material for: Endothelin-1 induces Zfp36 family RNA-binding proteins and restrains cytokine and chemokine production in reactive astrocytes
Source: J Biol Chem. 2026 Apr 2;302(5):111424. doi: 10.1016/j.jbc.2026.111424 (PMC13129385; doi:10.1016/j.jbc.2026.111424)
Supplement: Supporting information [file mmc1.docx]

**Endothelin-1 induces Zfp36 family RNA binding proteins and restrains cytokine and chemokine production in reactive astrocytes**

Yutaka Koyama, Aina Nishiuma, Nagi Takahashi, Eri Izumikawa, Chisato Hamada, Yasuhiko Izumi, Shigeru Hishinuma and Shotaro Michinaga

**Supplementary information included:**

·Supplemental Figure S1 p2

·Supplemental Figure S2 p3

·Supplemental Figure S3 p4

·Supplemental Figure S4 p5

·Supplemental Figure S5 p6

·Supplemental Figure S6 p7

·Supplemental Table S1 p8-9

·Supplemental Table S2 p10

·Uncropped immunoblots of TTP, BRF-1 and β-actin. p11

·Immunoblots used for quantification p12-14

·Uncropped agarose gel electrophoresis images p15

**
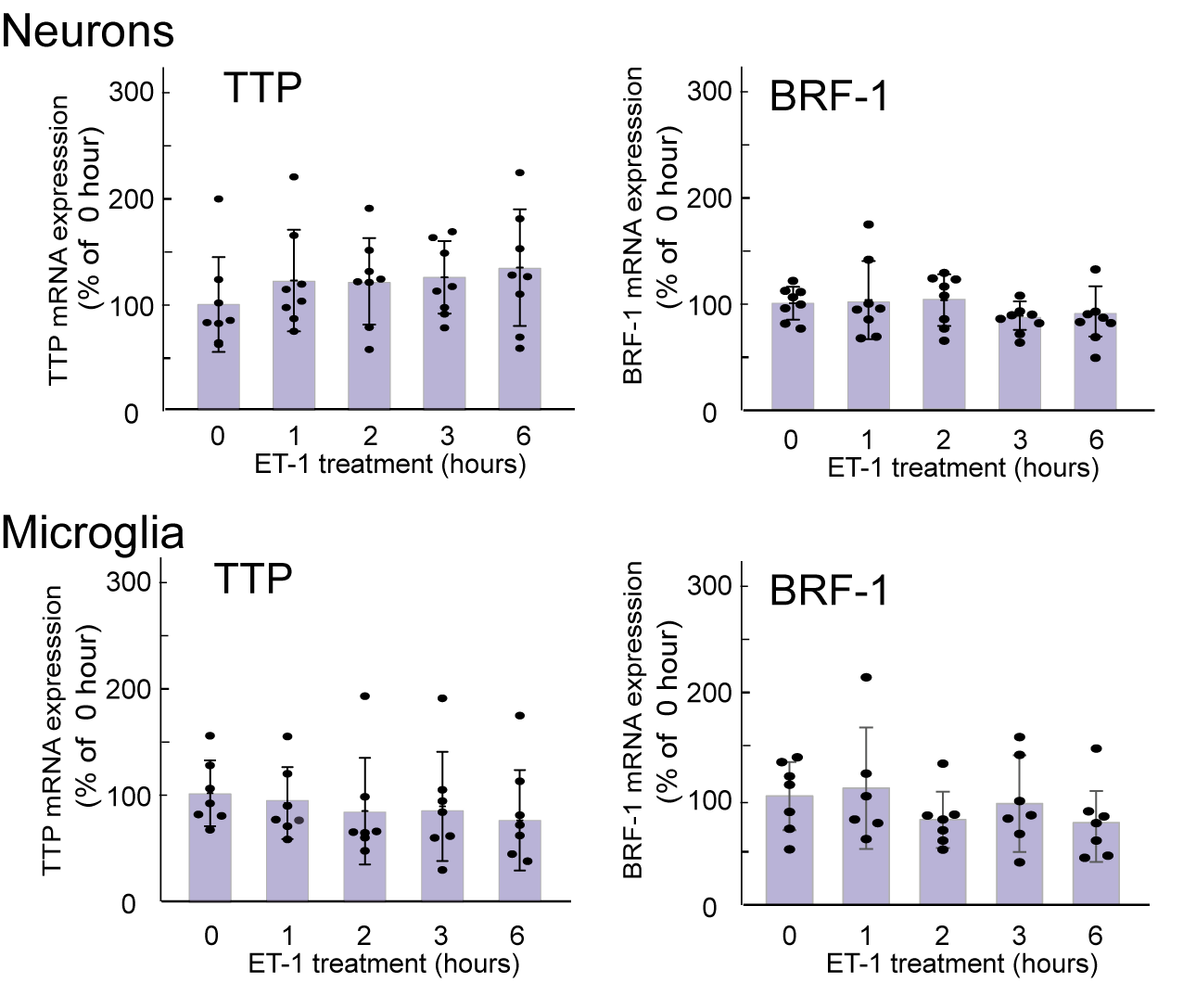
**

**Supplemental Figure S1. Effects of ET-1 on TTP and BRF-1 mRNA expressions in cultured rat cerebral neurons and microglia**. Cultured neurons and microglia are treated with 100 nM ET-1 for the indicated time. The expression of TTP and BRF-1 mRNAs are normalized to that of G3PDH. Results are presented as mean ± SD of eight and seven different mRNA preparations for neurons and microglia, respectively. Individual data points are indicated by dots.


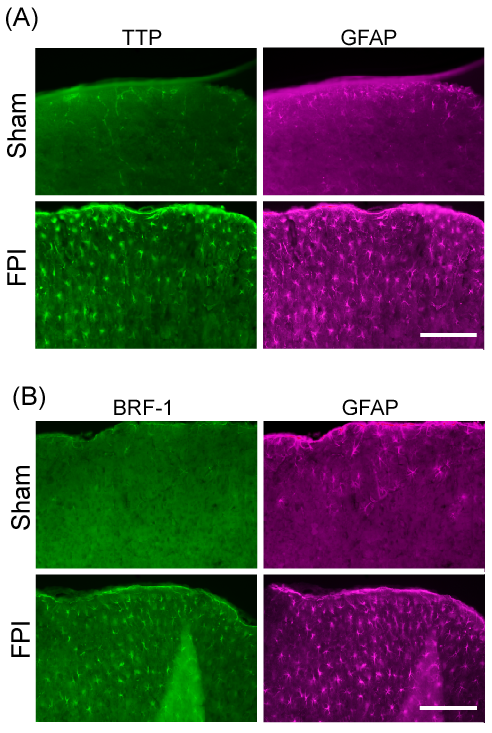


**Supplemental Figure S2.**  **Immunohistochemical observation of TTP and BRF-1 in the cerebrum of sham-operated or FPI mice.** Two days after FPI, TTP (A) and BRF-1 (B) expression in the injured area of mouse brain was observed by a double-labeling with anti-GFAP antibody. For sham-operated mice, the same cerebral area as that of FPI mice was observed. Scale bar = 200 μm.


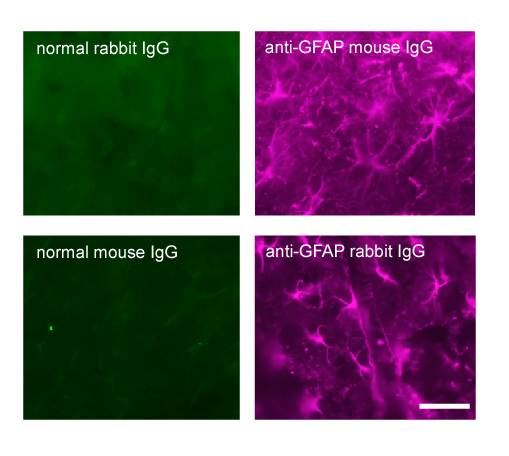


**Supplemental Figure S3.**  **Negative control staining of double-labelling with anti-GFAP antibodies in the mouse cerebrum.** Two days after FPI, mouse brains were fixed by 4% paraformaldehyde and frozen sections were prepared. Brain sections were incubated with anti-GFAP rabbit IgG (cat#12389, Cell Signaling Tech.) or mouse IgG (cat#G3893, Sigma-Aldrich) antibody. As a negative control, normal mouse IgG (cat#sc-2025, St. Cruz) or rabbit IgG (cat#30000-0-AP, Proteintech) was used instead of antibodies against RBPs and chemokine/cytokine. In the negative control, GFAP-positive cells did not show obvious FITC-staining. Scale bars = 50 µm.

**
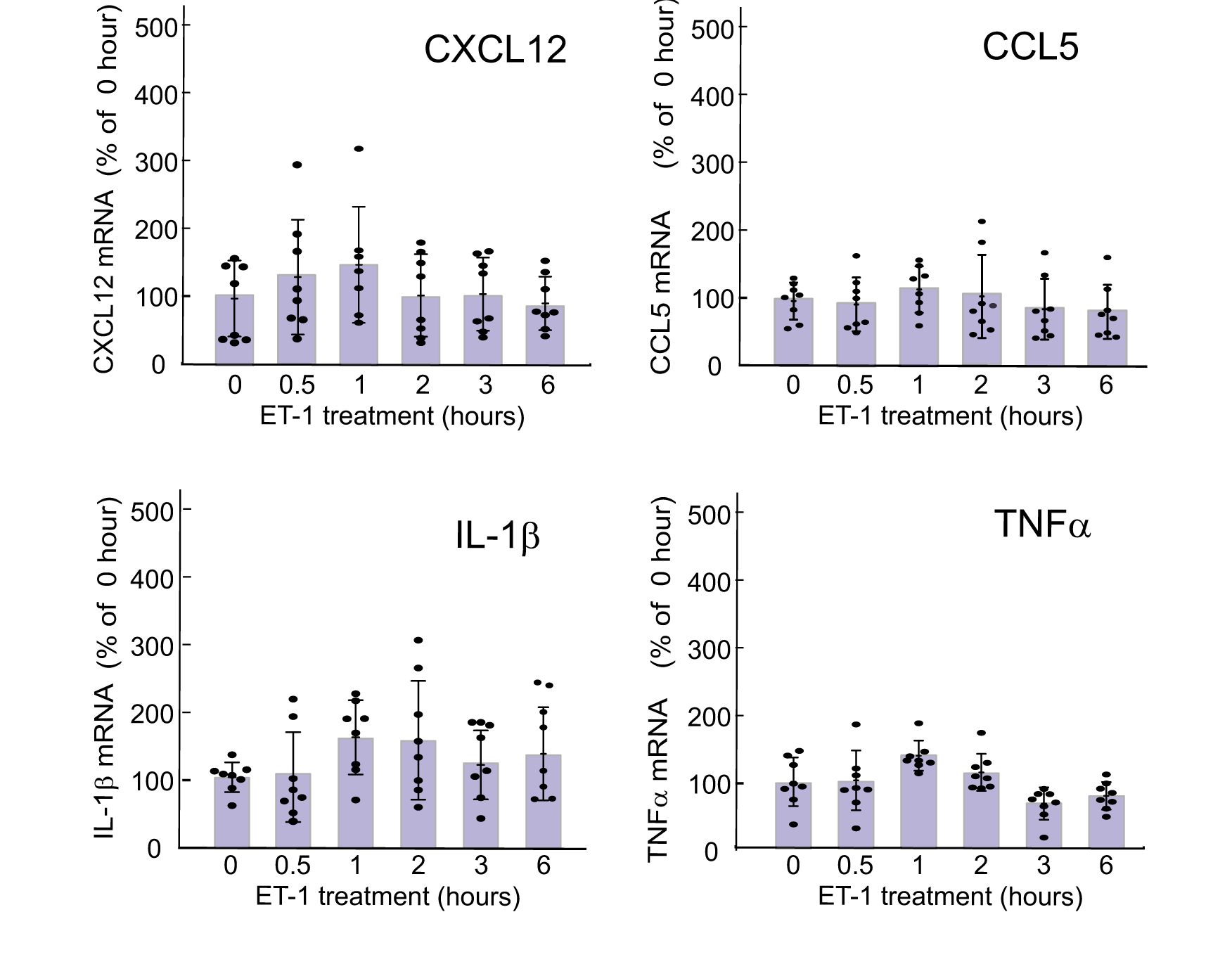
**

**Supplemental Figure S4. Effects of ET-1 on CXCL12, CCL5, IL-1β and TNFα mRNAs in cultured astrocytes**. Cultured astrocytes were treated with 100 nM ET-1 for the indicated time. The expression of chemokine/cytokine mRNAs were normalized to that of G3PDH. Results are presented as mean ± SD of eight and seven different mRNA preparations for neurons and microglia, respectively. Individual data points are indicated by dots.


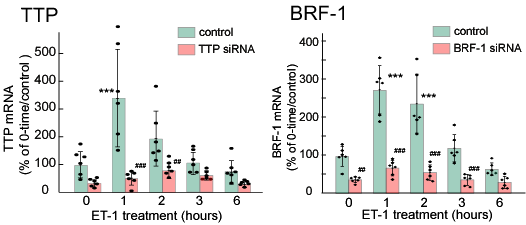


**Supplemental Figure S5. Knockdown efficacies of TTP and BRF-1 siRNA transfection:** After transfected with control or RBP siRNAs, cultured astrocytes are treated with 100 nM ET-1 for 1 and 6 hours. The expression levels of TTP and BRF-1 mRNA were normalized to that of G3PDH. Results are presented as mean ± SD of six different mRNA preparations. Shapiro–Wilk test showed that the data for all groups were normally distributed (*p* = 0.1990 to 0.9833). Therefore, the effects of siRNA were analyzed by two-way ANOVA [TTP; *F*(1,50) = 45.20, *p* = 1.623×10^-8^, BRF-1; *F*(1,50) = 142.2, *p* = 3.077×10^-16^]. ****p* < 0.001 vs. no ET-1 treatment (0 hour), ^~~##~~^*p* < 0.01, ^~~###~~^*p* < 0.001 vs. each control by Tukey’s test**.**


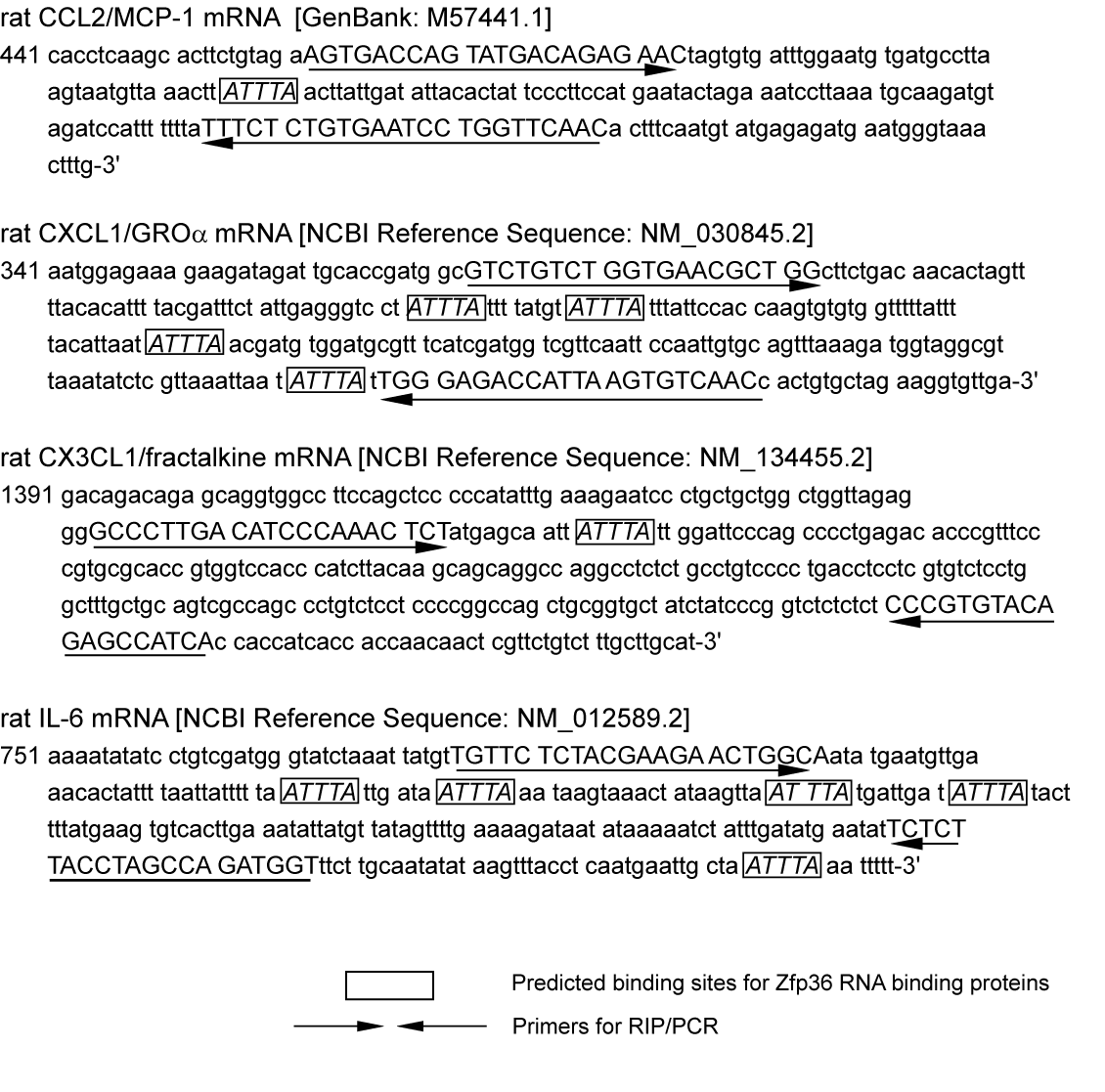


**Supplemental Figure S6. Primer sequences for RIP/PCR.**  Reported cDNA sequences for the 3’-flanking region of rat CCL2, CXCL1, CX3CL1 and IL-6 mRNAs are shown. Predicted binding sites for Zfp36 family RNA binding proteins are boxed. Each primer pair used for the RIP/PCR assay was designed from cDNA sequences indicated by arrows.

**Supplemental Table S1. Sequences of PCR primers**

**Primers for RBPs and chemokines/cytokines**

|  | Forward | Reverse |
| --- | --- | --- |
| rat TTP/Zfp36 | 5’- CTCAGAGAG CGGGCGTTGT -3’ | 5’- GATTGGCTTGGC GCAGTTCA -3’ |
| rat BRF-1/Zfp36L1 | 5’- TGGACAACTCAAGACGCCTG -3’ | 5’- CCTCCCTACCCTGGCTTAGT -3’ |
| rat BRF-2/Zfp36L2 | 5’- CGCTGGTCAACAAGGAAAGC -3’ | 5’- TGTAGCGCGTGGAGTTGATT -3’ |
| rat AUF-1 | 5’-GGGGACGTTGTAGACTGCACTCTGA-3’ | 5’- ACAAAGCCAAAACCCCTTGA -3’ |
| rat HuR | 5’- ACATCAGTGGGCTTCCAAGG -3’ | 5’- TGATTCGCCCAAACCGAGAA -3’ |
| rat KSRP | 5’- GAGGCATCGATGTGCCTGTA -3’ | 5’- GGTCCCGTCATCTTGCTTGA -3’ |
| mouse TTP/Zfp36 | 5’- CATGTGCTGCGACAAAGCAT -3’ | 5’- TGGGGGTAGTAGACCTTCGG -3’ |
| mouse BRF-1/Zfp36L1 | 5’- CACCAATAACCCCTTCGCCT -3’ | 5’- CTGCTCAGATAGCCCTCGTG -3’ |
| mouse BRF-2/Zfp36L2 | 5’- CACGCTTGCAATTTCGACCA -3’ | 5’- TTGCCAGGGATTTCTCCGTC -3’ |
| mouse AUF-1 | 5’- CAGAGGGAGCCAAGATCGAC -3’ | 5’- CTTCAGTGTGTCGTGGGGAG -3’ |
| mouse HuR | 5’- CCAACACCTTACCCCTTCCC -3’ | 5’- ATCCCTCCCCAGGAGTTACC -3’ |
| mouse KSRP | 5’- CAACTGGGACCCATCCATCC -3’ | 5’- CTCCAGTCAGAGACACGCTG -3’ |
| rat CCL2/MCP-1 | 5’- GCAGGTCTCTGTCACGCTTT -3’ | 5’- TGAGTGGGGCATTAACTGCAT -3’ |
| rat CXCL1/ GROα | 5’- ACTCAAGAATGGTCGCGAGG -3’ | 5’- ACGCCATCGGTGCAATCTAT -3’ |
| rat CX3CL1/  fractalkine | 5’- GAATTCCTGGCGGGTCAGCACCTC  GGCATA -3’ | 5’- AAGCTTTTACAGGGCAGCCGTCT  GGTGG -3’ |
| rat /SDFα | 5’- GCATCAGTGACGGTAAGCCA -3’ | 5’- AAGGGCACAGTTTGGAGTGT -3’ |
| rat /RANTES | 5’- CATATGGCTCGGACACCACT -3’ | 5’- TTCTTCTCTGGGTTGGCACA -3’ |
| rat preproET-1 | 5’- CGCTTCGCTCCGGTGAA -3’ | 5’- CTGAGCTCTGCTCCCAAGAC -3’ |
| rat IL-1β | 5’- AGCTTCAGGAAGGCAGTGTC -3’ | 5’- TCAGACAGCACGAGGCATTT -3’ |
| rat IL-6 | 5’- CACTTCACAAGTCGGAGGCT -3’ | 5’- TCTGACAGTGCATCATCGCT -3’ |
| rat TNFα | 5’- GGCTTTCGGAACTCACTGGA -3’ | 5’- GGGAACAGTCTGGGAAGCTC -3’ |
| mouse CCL2/MCP-1 | 5’- GACCCCAAGAAGGAATGGGT -3’ | 5’- ACCTTAGGGCAGATGCAGTT -3’ |
| mouse CXCL1/ GROα | 5’- GGTGTCCCCAAGTAACGGAG -3’ | 5’- TTGTCAGAAGCCAGCGTTCA -3’ |
| mouse CX3CL1/  fractalkine | 5’- AACTTCCGAGGCACAGGATG -3’ | 5’- AGATGTCAGCCGCCTCAAAA -3’ |
| mouse preproET-1 | 5’- TTCTTGCCGGTTGGGAATGA -3’ | 5’- TTTCTACAGAAACCCCGCCC -3’ |
| mouse IL-6 | 5’- ACAAGTCCGGAGAGGAGACT -3’ | 5’- TTCTGCAAGTGCATCATCGTT -3’ |
| G3PDH,  (rat & mouse) | 5’-aacgaccccttcattgacc-3’ | 5’- TCCACGACATACTCAGCAC -3’ |

**Primers for RNA-IP/PCR**

|  | Forward | Reverse |
| --- | --- | --- |
| CCL2/MCP-1 | 5’- AGTGACCAGTATGACAGAGAACT-3’ | 5’-GTTGAACCAGGATTCACAGAGAAA-3’ |
| CXCL1/ GROα | 5’- GTCTGTCTGGTGAACGCTGG -3’ | 5’-GTTGACACTTAATGGTCTCCCA-3’ |
| CX3CL1/  fractalkine | 5’- GCCCTTGACATCCCAAACTCT -3’ | 5’- TGATGGCTCTGTACACGGGA -3’ |
| IL-6 | 5’- TGTTCTCTACGAAGAACTGGCA -3’ | 5’-ACCATCTGGCTAGGTAAGAGA -3’ |

**Supplemental Table S2. Primary antibodies used for immunohistochemical observation**

|  |  | Supplier |
| --- | --- | --- |
| TTP | anti-ZFP36 rabbit polyclonal antibody | 12737-1-AP,  Proteintech, IL, USA |
| BRF1 | anti-TFIIIB90-1/2/3/5 mouse monoclonal  antibody, clone A-8 | sc-390821,  Santa Cruz Biotech, CA, USA |
| CCL2 | anti-MCP-1/CCL2 rabbit polyclonal antibody | 26161-1-AP,  Proteintech, IL, USA |
| CXCL1 | anti-CXCL1 rabbit polyclonal antibody | 12335-1-AP,  Proteintech, IL, USA |
| IL-6 | anti-IL-6 mouse monoclonal antibody,  clone 10E5 | sc-57315,  Santa Cruz Biotech, CA, USA |
| ET-1 | anti-endothelin-1 mouse monoclonal antibody,  clone C-7 | sc-517436,  Santa Cruz Biotech, CA, USA |
| ALDH1L1 | anti-ALDH1L1 rabbit polyclonal antibody, | 17390-1-AP,  Proteintech, IL, USA |

**
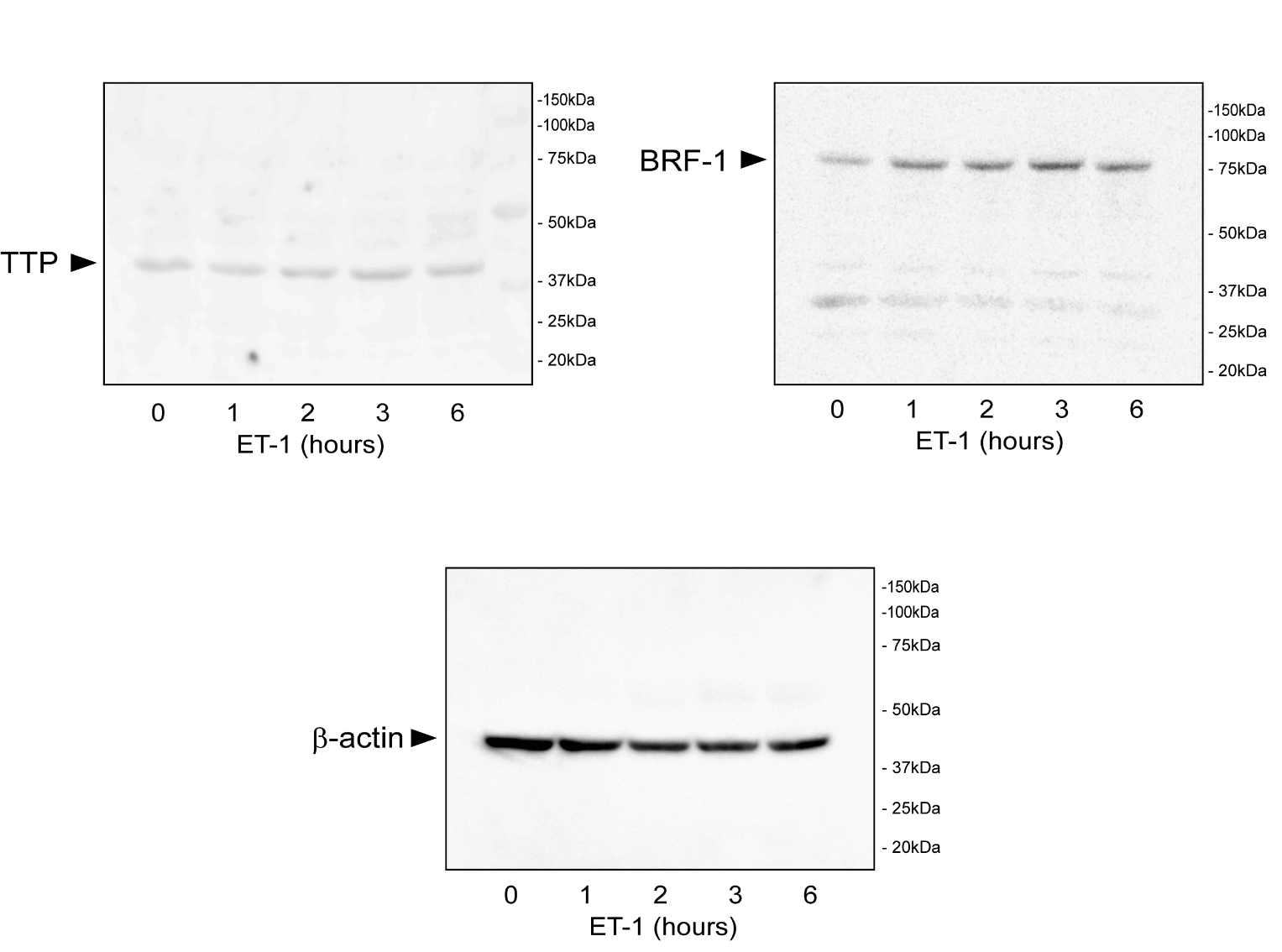
**

**Uncropped immunoblots of TTP, BRF-1 and β-actin**.

Cultured astrocytes were treated with 100 nM ET-1 for the indicated times. Representative uncropped immunoblots of TTP, BRF-1 and β-actin electrophoresed on 10% acrylamide gel are shown. Precision Plus Protein™ Dual Xtra Prestained Protein Standards (cat#1610377, Biorad) was used for a molecular weight marker.


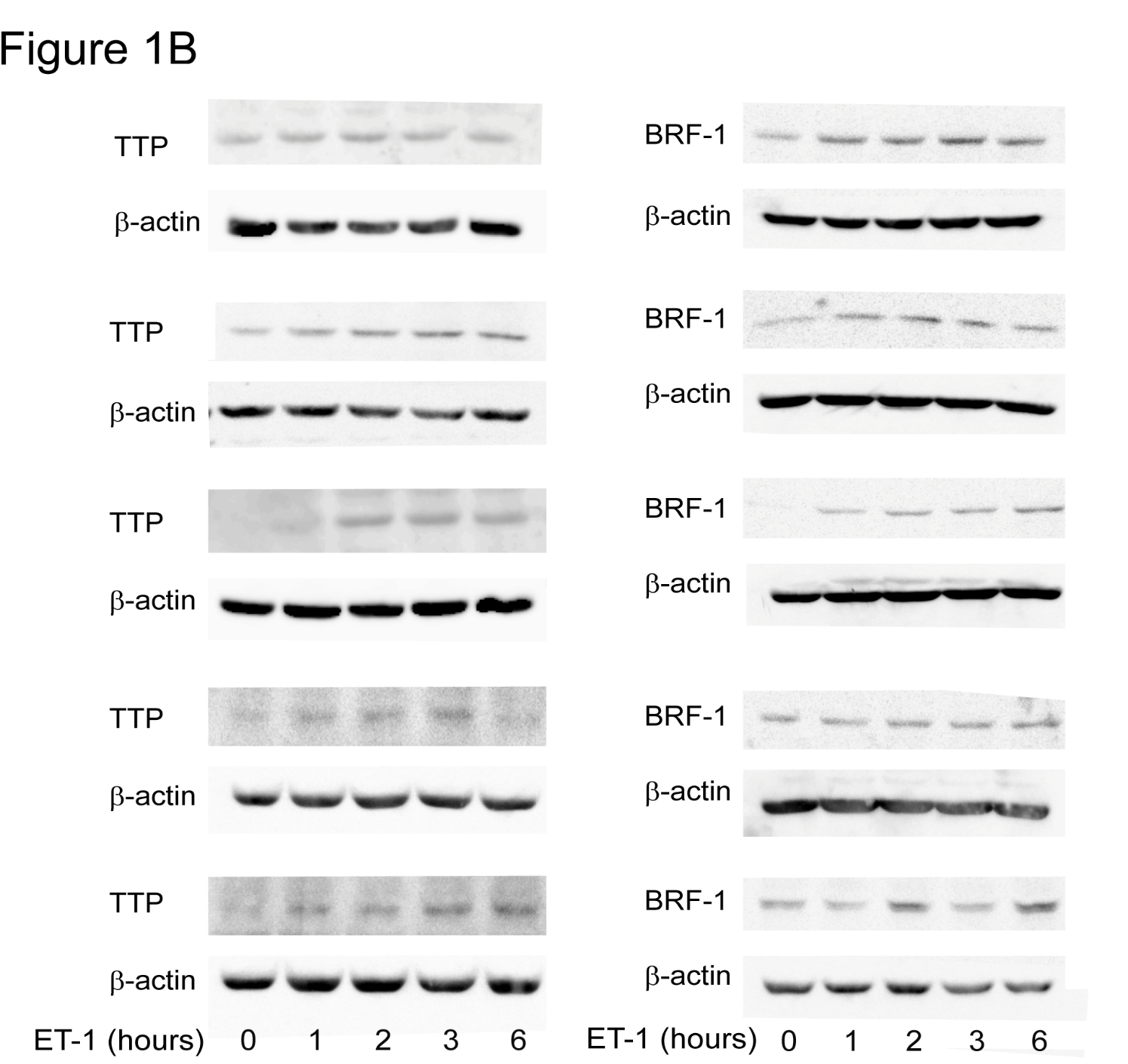


**Immunoblots used for quantification in Figure 1B**

Cultured astrocytes were treated with 100 nM ET-1 for 1-6 hours. Five different protein preparations were subjected to immunoblot analysis. Density of the protein bands was quantified using ImageJ 1.45 software (US. NIH, Bethesda, Maryland, USA) and the results were given in Figure 1B.

**
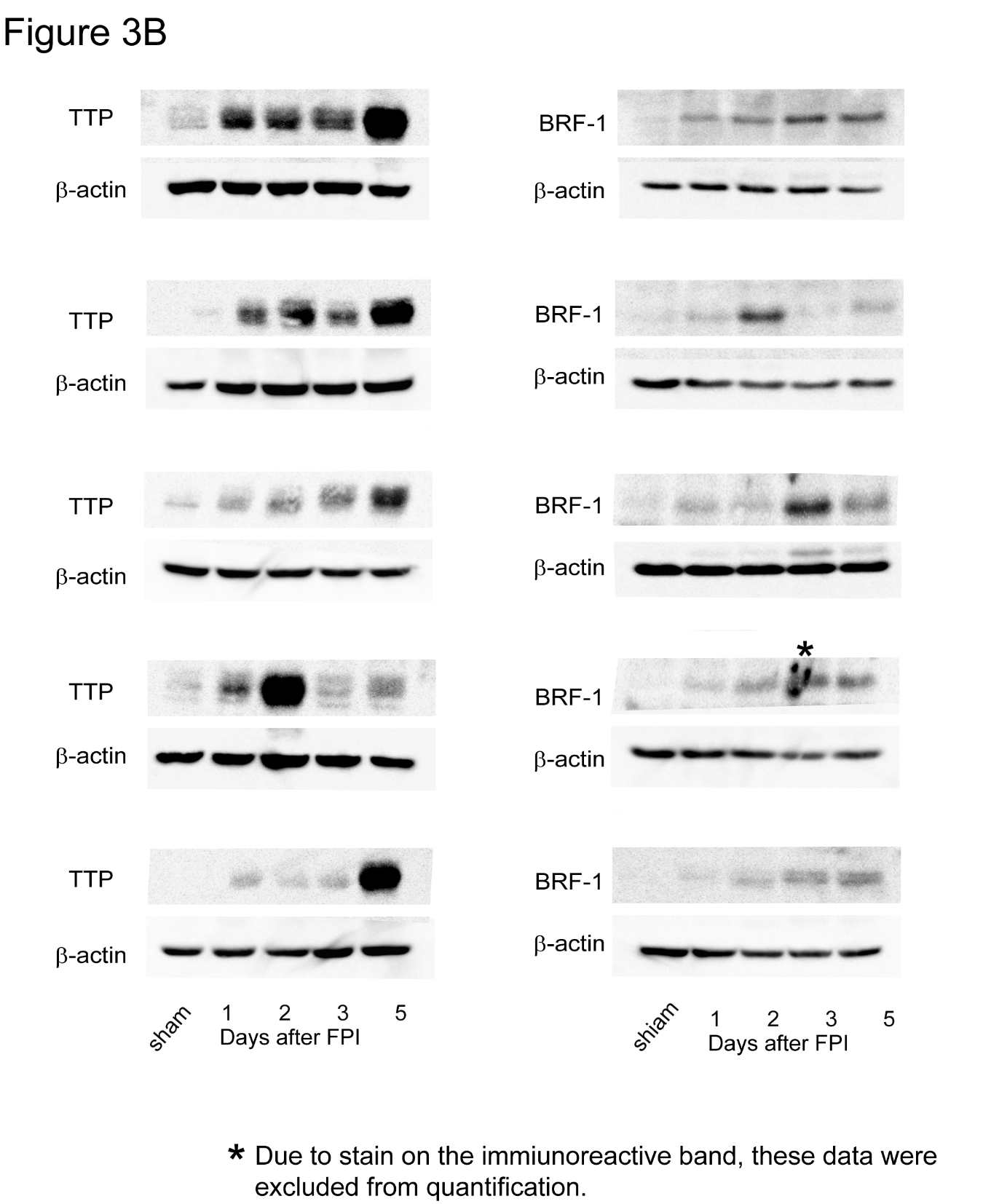
**

**Immunoblots used for quantification in Figure 3B**

Five mice were subjected to FPI for each condition. Protein preparation from each mouse was subjected to immunoblot analysis. Density of the protein bands was quantified using ImageJ 1.45 software (US. NIH, Bethesda, Maryland, USA) and the results were given in Figure 3B.


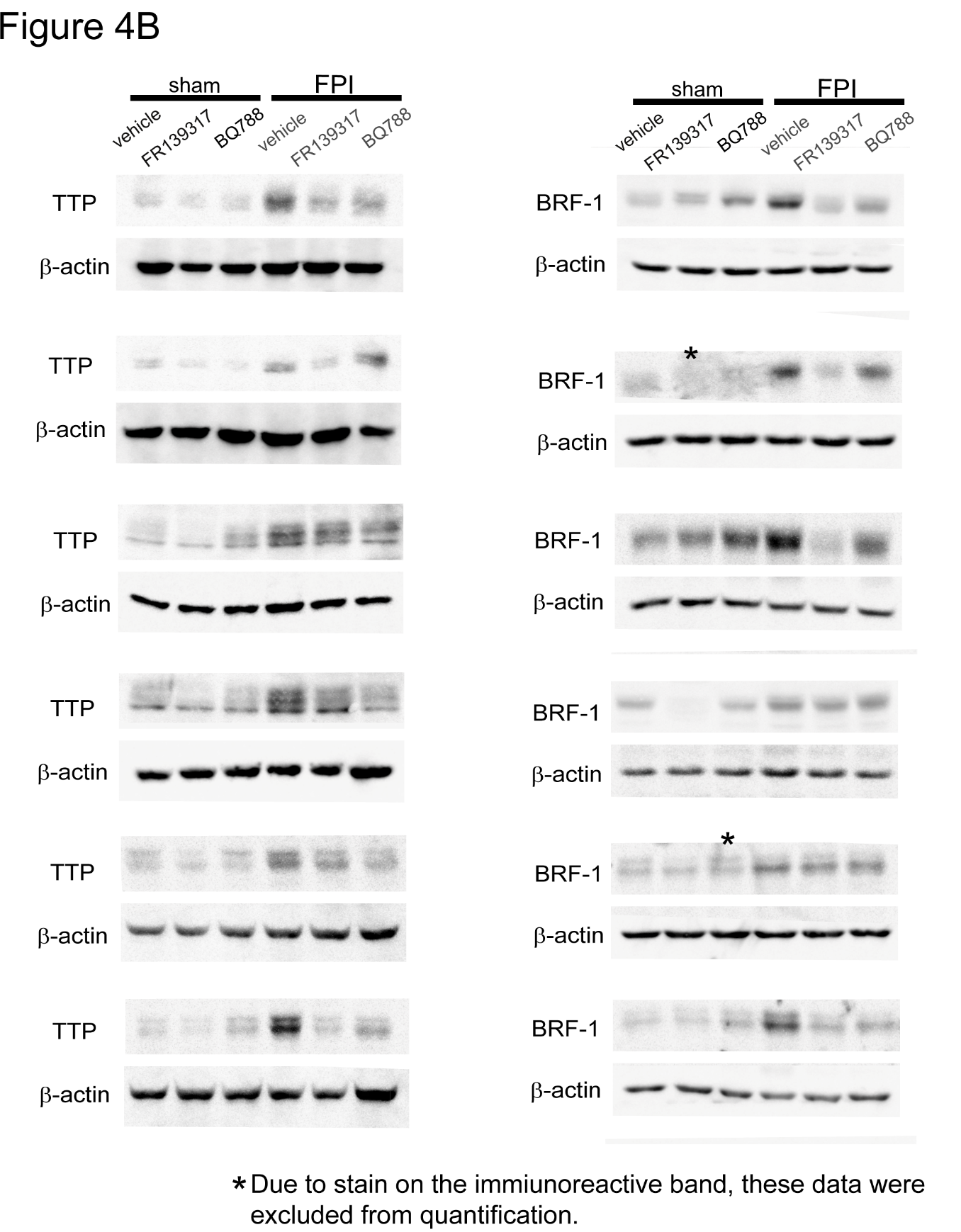


**Immunoblots used for quantification in Figure 4B**

Six mice were subjected to FPI for each condition. Protein preparation from each mouse was subjected to immunoblot analysis. Density of the protein bands was quantified using ImageJ 1.45 software (US. NIH, Bethesda, Maryland, USA) and the results were given in Figure 4B.


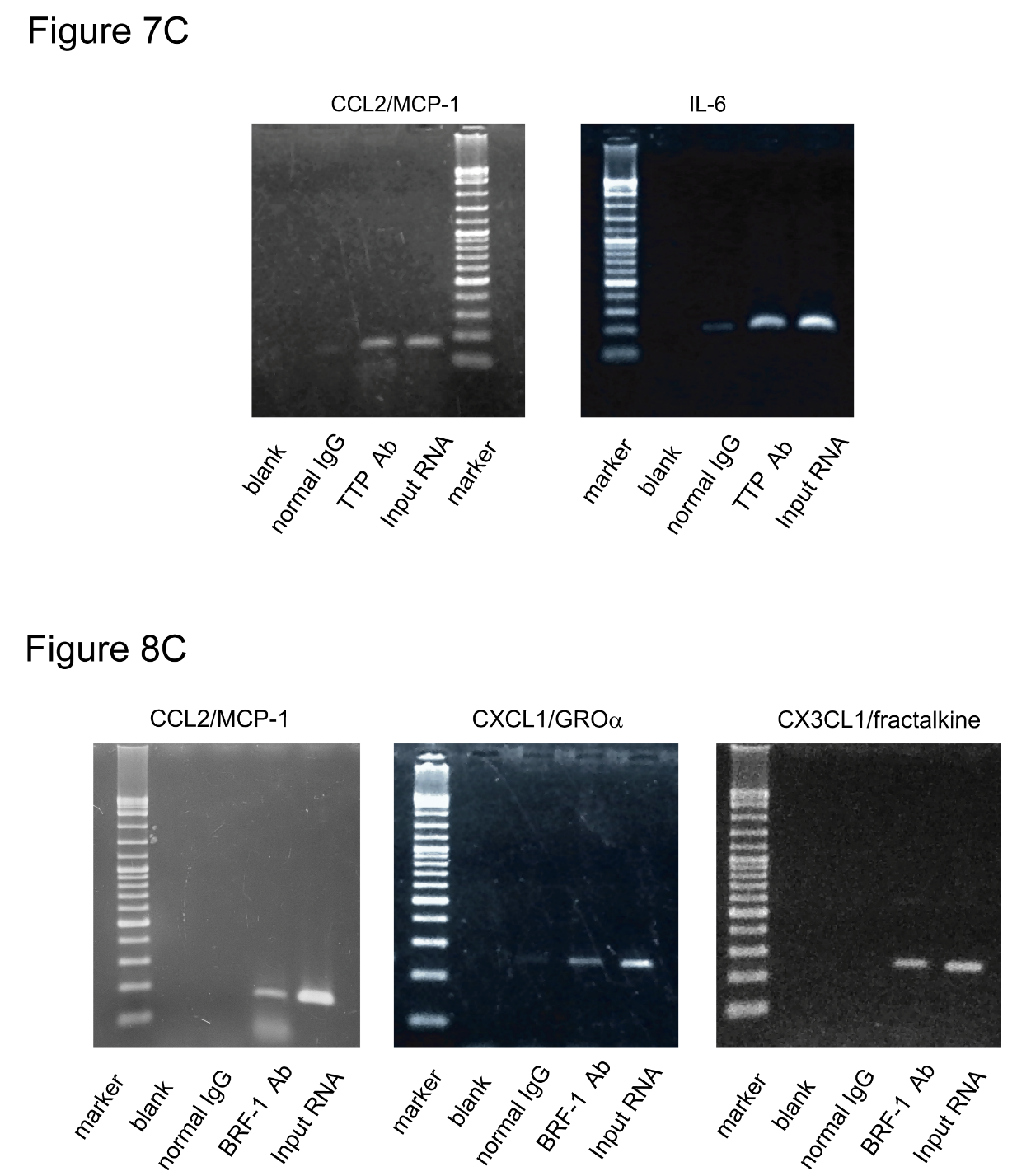


**Uncropped agarose gel electrophoresis images in Figure 7C and 8C**.

The PCR products were electrophoresed in 2% agarose gel and stained with 1 μg/mL ethidium bromide. DNA Ladder One™ (cat# 07908-75, Nacalai Tesque Inc.) was used for a molecular weight marker.
